# Biomimetic hydrogel blanket for conserving and recovering intrinsic cell properties

**DOI:** 10.1186/s40824-022-00327-w

**Published:** 2022-12-13

**Authors:** Seung-Hoon Um, Youngmin Seo, Hyunseon Seo, Kyungwoo Lee, Sun Hwa Park, Jung Ho Jeon, Jung Yeon Lim, Myoung-Ryul Ok, Yu-Chan Kim, Hyunjung Kim, Cheol-Hong Cheon, Hyung-Seop Han, James R. Edwards, Sung Won Kim, Hojeong Jeon

**Affiliations:** 1 Biomaterials Research Center, Biomedical Research Division, Korea Institute of Science and Technology (KIST), 02792 Seoul, Republic of Korea; 2 Laboratory for Biomaterials and Bioengineering, Department of Min-Met-Materials Engineering, Research Center of CHU de Quebec, Division of Regenerative Medicine, Canada Research Chair I in Biomaterials and Bioengineering for the Innovation in Surgery, Laval University, G1V 0A6 Quebec City, Quebec, Canada; 3 R&D Institute, OID Ltd, Seoul, 06286 Republic of Korea; 4 School of Medicine, Sungkyunkwan University, Suwon, 16419 Republic of Korea; 5 Department of Otolaryngology-Head and Neck Surgery, College of Medicine, The Catholic University of Korea, Seoul, Republic of Korea; 6 Department of Biomedicine & Health Sciences, Department of Otolaryngology-Head and Neck Surgery, College of Medicine, The Catholic University of Korea, Seoul, Republic of Korea; 7 Division of Bio-Medical Science and Technology, KIST School, Korea University of Science and Technology, Seoul, 02792 Republic of Korea; 8 Division of Nursing, Research Institute of Nursing Science, Hallym University, Chuncheon, 24252 Republic of Korea; 9 Department of Chemistry, Korea University, Seoul, 02841 Republic of Korea; 10 Nuffield Department of Orthopaedics, Rheumatology and Musculoskeletal Sciences (NDORMS), Botnar Research Centre, University of Oxford, Oxford, OX3 7LD UK; 11 KU-KIST Graduate School of Converging Science and Technology, Korea University, Seoul, 02841 Republic of Korea

**Keywords:** Biomimetic, Hydrogel blanket, Cell culture system, Physical stimuli, Conserving and recovering cell properties

## Abstract

**Background:**

Cells in the human body experience different growth environments and conditions, such as compressive pressure and oxygen concentrations, depending on the type and location of the tissue. Thus, a culture device that emulates the environment inside the body is required to study cells outside the body.

**Methods:**

A blanket-type cell culture device (Direct Contact Pressing: DCP) was fabricated with an alginate-based hydrogel. Changes in cell morphology due to DCP pressure were observed using a phase contrast microscope. The changes in the oxygen permeability and pressure according to the hydrogel concentration of DCP were analyzed. To compare the effects of DCP with normal or artificial hypoxic cultures, cells were divided based on the culture technique: normal culture, DCP culture device, and artificial hypoxic environment. Changes in phenotype, genes, and glycosaminoglycan amounts according to each environment were evaluated. Based on this, the mechanism of each culture environment on the intrinsic properties of conserving chondrocytes was suggested.

**Results:**

Chondrocytes live under pressure from the surrounding collagen tissue and experience a hypoxic environment because collagen inhibits oxygen permeability. By culturing the chondrocytes in a DCP environment, the capability of DCP to produce a low-oxygen and physical pressure environment was verified. When human primary chondrocytes, which require pressure and a low-oxygen environment during culture to maintain their innate properties, were cultured using the hydrogel blanket, the original shapes and properties of the chondrocytes were maintained. The intrinsic properties could be recovered even in aged cells that had lost their original cell properties.

**Conclusions:**

A DCP culture method using a biomimetic hydrogel blanket provides cells with an adjustable physical pressure and a low-oxygen environment. Through this technique, we could maintain the original cellular phenotypes and intrinsic properties of human primary chondrocytes. The results of this study can be applied to other cells that require special pressure and oxygen concentration control to maintain their intrinsic properties. Additionally, this technique has the potential to be applied to the re-differentiation of cells that have lost their original properties.

**Supplementary Information:**

The online version contains supplementary material available at 10.1186/s40824-022-00327-w.

## Background

The human body consists of cells [1]. Cells derived from the human body allow assessments to be conducted without threatening the person’s life [2]. Given that cells live in different environments depending on the function and location of the tissue from which they are derived [3], they lose their intrinsic properties when their environment differs from their original growth environment [4]. Cells that lose intrinsic properties respond differently to chemical and physical stimuli than normal cells [5]. Therefore, for a more accurate evaluation, a culture technology that can mimic the original growth environment is essential [6]. Nutrients [7, 8], proteins [9], inorganic ions [10], etc. in the body environment can be mimicked with a cell culture medium, even in conjunction with the use of a general culture device [11]. However, to control the conditions of the internal physiological environment, such as nitrogen, carbon dioxide, oxygen concentration, and pressure, special culture equipment is required, such as a compressive pressure control device [12], oxygen concentration control accessories [13], an electrochemical stimulation apparatus [14], sandwich culture device [15], three-dimensional (3D) culture appliances using hydrogel [16], and bioreactors [17]. However, it costs approximately 17,000 USD or more to equip an appropriate cultural appliance [18] and pressure chamber [19], which is a significant economic burden. Furthermore, there is a space constraint pertaining to the installation of new equipment because the existing incubator cannot be utilized [20]. Conversely, 3D culture methods using hydrogels are relatively inexpensive and impose smaller space requirements. However, considering that cells are cultured in a hydrogel, it is difficult to recover pure cells after culturing [21]. Despite the efforts expended to emulate the internal environment of the human body, it has been challenging to maintain the intrinsic properties of cells, and the reason for this is also uncertain [22].

In this study, a commercially available cell culture insert was used, and a hydrogel sheet was formed on its bottom part to fabricate a facile tool for the design of a stamp-like layer for approximately 8 USD [23]. This method is referred to as a direct contact pressing (DCP) culture with a biomimetic hydrogel blanket because the hydrogel directly covers the cells like a stamp, thus creating pressure and a hypoxic environment. The hydrogel blanket has a relatively small size (approximately 30 mm in diameter and 15 mm in height) and can be directly applied to a general cell culture dish without a separate uncommon culture device. Furthermore, as the cells are covered with the hydrogel, it is possible to induce specific cell pressures, such as in the case of 3D cultures [21]. Chondrocytes live under pressure from the surrounding collagen tissue and experience a hypoxic environment of approximately 1% because collagen inhibits oxygen permeability [24]. Likewise, this DCP culture can apply pressure directly by covering the cells with the hydrogel and by realizing a low-oxygen environment, which is achieved by inhibiting oxygen diffusion without the need for a separate external device. Moreover, the pressure and oxygen concentration applied to the cells can be regulated by the amount of solution used to fill the hydrogel blanket reservoir and by the concentration of the hydrogel. Human primary chondrocytes lose their original properties without adequate pressure and a hypoxic environment during incubation [25]. These chondrocytes were cultured in a hydrogel blanket environment to check whether their intrinsic properties were maintained. This was to verify whether the hydrogel blanket could provide a pressure and low-oxygen environment similar to the environment in the body. Based on these efforts, it was ascertained whether it is possible to culture human primary chondrocytes while maintaining their intrinsic properties by only using the hydrogel blanket in a general cell incubator. To confirm the efficacy of the hydrogel blanket, cell morphology [26], gene expression [27], and glycosaminoglycan (GAG) [28] contents were studied as these are known to be affected by the intrinsic properties of cells. It was also determined whether the hydrogel blanket influenced the recovery of the intrinsic properties even when the cells lost their original properties. Ultimately, a mechanism was suggested for the conservation and recovery of the original properties of cells cultured with the hydrogel blanket without external equipment based on signaling pathway analysis. In other words, we provide a technology to maintain the original intrinsic properties of cells that require specific environments such as pressure and hypoxia. In addition, this study has the potential to provide fundamental insights into the rejuvenation of cells that have lost their original properties.

## Methods

### Hydrogel blanket preparation

Sodium alginate (9005–38-3, Sigma-Aldrich) was mixed with distilled water to a concentration of 2 wt% and stirred at room temperature for 12 h to prepare alginate solution. Dimethyl siloxane, Dimethylvinylsiloxy-terminated (SYLGARD 184 Silicone elastomer base, Dow) and SYLGARD 184 Silicone elastomer agent (Dow) were mixed at a ratio of 1: 8, and then a plastic plate having a diameter of 15 cm (CLS430599, Corning) was poured to a thickness of 1 cm, after putting it in the chamber (Kartel 243,065, Dynalon), the vacuum pump (DOA-P504, Gast) was operated and kept at a low pressure of -740 mm Hg or more for 1 h to remove air bubbles. Followed by curing in a 70℃ oven for 24 h to produce a polydimethylsiloxane (PDMS) plate. The alginate solution 400 μl was poured onto the PDMS plate and then gently covered with a cell culture insert (PICM03050, Millipore) of 30 mm in diameter so that the solution became a disc shape. Then, 1 M CaCl_2_ (10,043–52-4, Sigma-Aldrich) solution was reacted with alginate solution in a disk form for 1 min to prepare alginate hydrogel. The gelled alginate was immersed in phosphate buffered saline (P5368, Sigma-Aldrich) solution to remove excess calcium ions, and the solution was changed once a day for a total of 3 days, and stored at 4℃ until the experiment. Alginate hydrogel was immersed in Dulbecco's Modified Eagle Medium (DMEM, Gibco) 3 h before cell experiments and UV sterilized for 1 h on a clean bench.

### Oxygen permeability measurement

Oxygen permeability according to the alginate gel concentration of the hydrogel blanket was measured. Seven types of specimens were prepared to measure the oxygen permeability: a cell culture insert without membrane, a cell culture insert with membrane only, and 5 cell culture inserts with 1, 2, 4, 6, and 8% alginate gel (*n* = 9, total = 63 ea). After preparing a 1L beaker (B3000-1L, Corning Pyrex) and filling 700 ml of distilled water. Oxygen meters (550A, YSI) were prepared to measure the amount of dissolved oxygen in water. Subsequently, the probe of the oxygen meter was immersed in the prepared water, waited for more than 30 s for stabilization, and measured. Distilled water was aliquoted to fill a 50 ml cortical tube (50,050, SPL). Reservoir was then placed on a cortical tube filled with water and sealed with a parafilm (PA 13,374, Parafilm M) for the water in the conical tube could only contact with the solution filled in the reservoir. At this time, reservoir prepared (i) without a membrane, (ii) with a membrane only, and a (iii) membrane and an alginate gel blanket together. In addition, the composition of alginate gel was prepared at 1, 2, 4, 6, and 8%, and the effect of oxygen permeation according to the gel concentration was also measured. A total of 63 samples of 7 types were maintained for 48 h at 37 °C, 5% CO_2_, 95% humidity, 1% O_2_, 94% N_2_ environment incubator (SMA-30DR, ASTEC). At 1 h, 3 h, 6 h, 9 h, 12 h, 24 h, and 48 h, the hydrogel blanket was taken out, and the oxygen concentration of distilled water in the reservoir and the conical tube was measured, respectively. The relative oxygen permeability (%) was calculated using the difference between the oxygen concentration in the reservoir and the conical tube.

### Compressive pressure measurement

The pressure applied by the hydrogel blanket tools to the cells was measured based on consideration of the total volume, buoyancy, and cell culture media density. First, a sample of the same conditions used in cell culture was prepared to measure the compressive pressure formed by the hydrogel blanket tool. This condition is filled with a total of 4 ml of cell culture media in the cell insert part of the hydrogel blanket. First, in order to measure the compressive pressure formed by the hydrogel blanket tool, a sample of the same environment was prepared during cell culture. This condition is to fill the cell insert part of the hydrogel blanket tool with a culture solution. The cell experiment was carried out by filling a total of 4 ml of cell culture fluid. Subsequently, the total mass of the hydrogel blanket tool filled with 4 ml of cell culture solution was measured. This is the total mass without considering the buoyancy of the hydrogel blanket tool. In order to obtain a buoyancy, the depth and area of the region where the hydrogel blanket tool is immersed in the solution were measured. The volume was multiplied by the density of the solution to obtain a buoyancy. Subsequently, the buoyancy was subtracted from the total mass of the hydrogel blank tool, and the pressure was obtained by dividing it by the hydrogel area. In addition, after preparing 2, 4, 6, and 8% hydrogel, the pressure of the hydrogel blanket tool according to each alginate composition was measured by measuring the same method as above.

### Direct contact pressing culture of chondrocyte using hydrogel blanket

Chondrocytes were seeded at 2.5 × 10^5^ cells / well in a 6-well plate (CLS3516, Corning) and cultured for 1 h (37℃, 5% CO_2_, 95% humidity) to allow cells to attach to the bottom of the plate. Subsequently, the prepared alginate hydrogel was placed directly on the cell so that it could be subjected to direct contact pressing. In the pressed state, culture was performed in normoxia and hypoxia environments according to the purpose of each experiment.

### Human primary chondrocyte cell culture

Human-derived nasal septal chondrocyte was provided by Prof. Seongwon Kim from the Catholic University of Korea for the experiment. The cell stock was dispensed into the freezing cell stock in DMEM containing 50% fetal bovine serum (FBS, Hyclon) and 10% dimethyl sulfoxide solution (DMSO, Sigam-Aldrich). The cell stock was stored in liquid nitrogen at -196℃ until the experiment, before the experiment, the cells were thawed in a 37℃ water bath and 30 ml of DMEM was added to remove the DMSO used for cell freezing through dilution, centrifugation and supernatant removal. The chondrocytes obtained from the nasal septum were regarded as passage 1, and when cultured in vitro, passage 2, 3 was named in order of passage. Chondrocytes were cultured in Dulbecco's Modified Eagle Medium (DMEM, Gibco) solution containing 10% fetal bovine serum (FBS, Hyclone) and 1% penicillin–streptomycin (Gibco) at a concentration of 2.5 × 10^5^ cells / well in a 6-well plate (Costar, Corning). Normoxia cultures were incubated at 37℃, 5% CO_2_, 95% humidity or more, in a 20% O_2_ environmental incubator (IR230, Thermo), and hypoxia cultivation was carried out in a common hypoxic environment of 37℃, 5% CO_2_, 95% humidity, 1% O_2_, 94% N_2_ environment (SMA-30DR, Astec).

### Gene expression analysis

The cultured media of chondrocytes cultured in each environment was removed and washed 3 times with phosphate buffered saline (PBS, pH 7.4, Gibco). RNA was extracted using an RNA extraction kit containing the QIAzol lysis reagent (RNeasay kit, Qiagen), and the amount of RNA was quantified using a nanodrop (ND-1000, NanoDrop). 300 ng or more per sample was used. The purified RNA obtained from chondrocyte was used at a concentration of 300 ng or more per sample. The purified RNA was collected in Maxime ™ RT PreMix (Intron) to 10 ng per sample and cDNA was synthesized using thermal cycler (Takara). Collagen type I (COL1), collagen type II (Col2), aggrecan (AGC), Sox9, hypoxia induced factor-1α (HIF-1α), transforming growth factor-β (TGF-β), SMAD family member 2 (Smad-2), SMAD family member 5 (Smad-5) and runt-related transcription factor 2 (Runx-2), each primer was designed (Table S3) using National Center for Biotechnology Information (NCBI, United States National Library of Medicine) gendat, the primer was manufactured through the company (COSMO) and then used. Polymerase chain reaction (PCR) was carried out using TB Green Premix kit (Takara) and analyzed using a PCR measuring device (7500 Real Time PCR System, Applied Biosystems). The relative expression level of the gene was compared with ‘normoxia control (NC)’ after normalization to the glyceraldehyde 3-phosphate dehydrogenase (GAPDH) value of each experimental group using the 2^−ΔΔCt^ method.

### Protein expression analysis

Chondrocytes were cultured in each environment, and then washed with PBS three times to remove the excess cell culture media. The cells were then lysed by adding Radioimmunoprecipitation assay buffer (RIPA lysis buffer, Biosesang) 300 μl / well and the amount of protein in each sample was quantitated with bicinchoninic acid assay kit (Pierce BCA Protein assay kit, Thermo). Sodium dodecyl sulfate–polyacrylamide gel electrophoresis (SDS-PAGE) was performed. The electrophoretic gel was transferred to polyvinylidene difluoride membrane (PVDF, Roche) and stained with ponceau-S solution (Sigma-Aldrich) to identify the protein bands and cut the protein positions to be identified. Each membrane was washed three times for 10 min with tris buffered saline buffer with Tween 20 (TBST, Biosesang) and then immersed in 3 wt / vol% skim milk (BD Difco) solution prepared with TBST for 1 h to prevent nonspecific binding. Subsequently, they were washed three times for 10 min each with TBST, and the primary antibody solution was added thereto, followed by reaction at room temperature for 1 h and 30 min and secondary antibody for 1 h and 30 min. After the antibody reaction was completed, the ECL solution (GE Healthcare amercham ECL select western blotting detection reagent RPN2235, GE) was applied to the membrane, and images were obtained by image equipment (LAS-3000, Fujifilm) and analyzed. For immunoassays collagen I antibody (ab34170, Abcam), collagen II antibody (ab34712, Abcam), aggrecan antibody (ab3778, Abcam), SOX9 antibody (ab185966, Abcam), HIF-1α (ab2185, Abcam), TGF beta 1 antibody (ab27969, Abcam), smad2 antibody (ab63576, Abcam), smad5 antibody (ab40771, Abcam) and Runx2 antibody (ab23981, Abcam) primary antibodies were used and for secondary antibodies HRP Goat Anti-Rabbit (IgG) secondary antibody (ab6721, Abcam) and HRP Rabbit Anti-Mouse (IgG) secondary antibody (ab6728, Abcam) were used.

### Glycosaminoglycans (GAGs) analysis

The amount of GAG in cultured chondrocytes in each environment was compared using Dimethylmethylene Blue (DMMB) [29, 30]. The chondrocytes cultured in each environment were washed three times with PBS to remove the excess cell culture media. 0.1 M sodium phosphate monobasic (S8282, Sigma-Aldrich) and 0.1 M sodium phosphate dibasic heptahydrate were mixed to prepare 0.1 M phosphate buffer (PB), and 10 mM Na-EDTA, 10 mM L-cysteine HCl, 25wt% papain were added to prepare a papain solution. The papain solution was dispensed at 0.5 ml / well to dissolve chondrocytes, and the amount of DNA contained in each sample was quantified by The PicoGreen assay (qQuant-IT PicoGreen dsDNA Reagent, Invitrogen), and the amount of GAG was measured using a calibration curve obtained by serial dilution of shark chondroitin sulfate (C4384, Sigma-Aldrich) in Dimethylmethylene blue (DMMB, 341,088, Sigma-Aldrich) solution. The measured values were expressed as GAG amount, DNA amount, and GAG / DNA amount.

### Cell morphology classification analysis

The shape of cultured chondrocytes in each environment was photographed using a phase contrast microscope (Nikon Eclipse TS100) and a CCD (HK5CCD-S, Sony). Each cell photograph was analyzed for cell length, width, and number of filopodia, lamellipodia using an image analysis program (Image J, NIH) and classified according to cell type classification criteria. Shape 1 was defined as a cell with a relatively round shape with two or fewer filopodia and lamellipodia, and a length-to-width ratio (a/b) < 5. The cells in which the length-to-width ratio is > 5, and the number of lamellipodia is ≥ 3, are classified as shape 3. A case in which the length-to-width ratio was < 5 and in which there were more than three filopodia was classified as shape 2. The percentage of cell form occupancy is expressed as a percentage of the number of cells of each type based on the total number of cells used for measurement in the image.

### Cell immunohistochemistry analysis

The morphology of chondrocytes and the expression of collagen type II and AGC were analyzed by immunohistochemistry. After culturing the chondrocytes in each environment, the culture medium was removed, and the media was rinsed three times with PBS to remove the excess media, and 4% paraformaldehyde solution was added and fixed for 20 min. Subsequently, the cell culture plate was rinsed three times with PBS, permeabilized with 0.5% Triton X-100 PBS solution for 5 min. After rinsing 3 times with PBS solution, 1% FBS solution was added and blocking was performed for 30 min. The cytoplasmic staining was performed with rhodamine phalloidin (R415, Invitrogen), and the primary antibody was collagen II antibody (ab34712, Abcam) and aggrecan antibody (ab3778, Abcam), respectively. Secondary antibodies were Alexa fluor 488 phalloidin (A12379, Invitrogen) and Alexa fluor 594 phalloidin (A12381, Invitrogen). All cells were stained with mounting medium for fluorescence with DAPI solution (H-1200, Vector) prior to imaging, and images were obtained by fluorescence microscopy (Imager A2m, ZEISS).

### Statistical analysis

All data were shown as the mean ± s.d. The obtained results were subjected to normality test using Kolmogorov–Smirnov. Analysis of variance (ANOVA) analysis was performed for normalization and Kruskal–Wallis analysis for nonparametric analysis. In the ANOVA analysis, the statistical significance of each group for one variable was analyzed by one-way ANOVA and the statistical significance of each group for two variables was analyzed by two-way ANOVA. After that, post hoc test was conducted with Tukey's multiple comparison. In box plot, each raw data is represented by dots, and mean and standard deviation are shown in box. And the maximum, minimum values are represented by whiskers. *p* < 0.05 is considered as statistically significant, and labeled as * *p* < 0.05, ** *p* < 0.01, *** *p* < 0.001 or # *p* < 0.05, ## *p* < 0.01 and ### *p* < 0.001. Each statistical significance test used a statistical analysis program (Origin 2020, Originlab).

## Results

### Preparation of biomimetic hydrogel blanket and effect on the cell culture environment

**Fig. 1:**
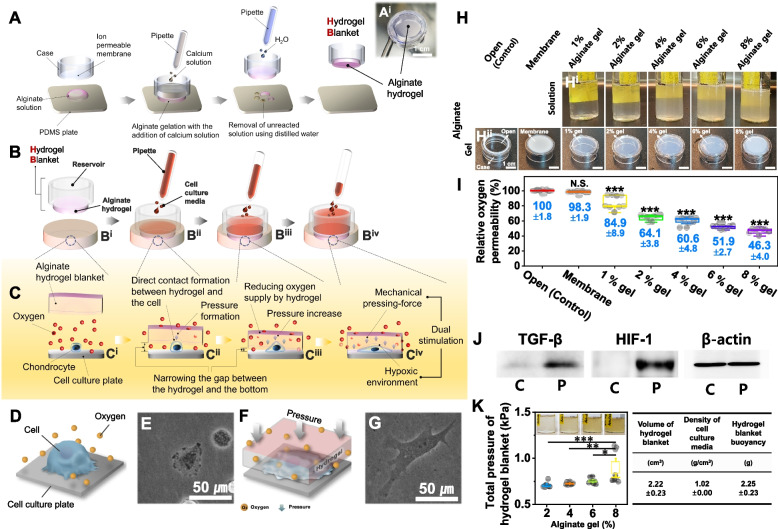
Fabrication process of biomimetic hydrogel blanket and the physicochemical effects on cell culture environment. (**A**) Schematic diagram of the manufacturing method of the hydrogel blanket and the picture of the manufactured hydrogel blanket (A^i^). Change of sedimentation depth of hydrogel blanket according to the amount of solution in the reservoir (**B**) and consequent cell-hydrogel blanket interaction diagram (**C**). Depiction of the environment of cells in culture without a hydrogel blanket (**D**) and observation result of their actual morphology using a phase-contrast microscope (**E**). The cultural environment diagram of the cells with the hydrogel blanket applied (**F**) and the consequent cell shape observed with a phase-contrast microscope (**G**). (**H**) 1, 2, 4, 6, 8 w/v % of alginate solution before reaction with calcium (Hi horizontal row). Image of the hydrogel blanket (alginate gel layer) formed on the membrane after reaction with 1 M calcium solution (Hii horizontal row). (**I**) The oxygen permeability of case without both gel and membrane (Control, Open), case with only membrane (Membrane), and the hydrogel blanket with membrane with gel of each concentration of 1, 2, 4, 6, 8%. (**J**) The results of western blot analysis of TGF-β, HIF-1, and β-actin expressed in cells when the hydrogel blanket environment of 2% gel concentration was applied to chondrocytes for 2 days. C (cell cultured without DCP, control), P (cell cultured with hydrogel blanket applied). (**K**) Total pressure according to the concentration of alginate used in the manufacture of hydrogel blanket. And the values used to calculate the total pressure (hydrogel blanket volume, buoyancy, and cell culture media density). ANOVA analysis and Tukey’s post-hoc test were performed for statistical significance for I and K, and the mean and standard deviation were presented as numerical values for I. N.S. (not significant), *P < 0.05, **P < 0.01, ***P < 0.001

To prepare the hydrogel blanket, calcium solution (1 M CaCl_2_), 2 weight/volume % alginate solution, and a cell culture insert (case) were used. The alginate solution was placed on a polydimethylsiloxane plate with a flat surface, the membrane—the central part of the case—was placed on top of the solution, and the calcium solution was poured and gelled to prepare the hydrogel blanket (Fig. 1A and Fig. S1). By changing the amount of cell culture media in the reservoir on top of the cell culture insert, the total mass of the hydrogel blanket could be controlled (Fig. 1B). In the absence of media in the insert reservoir, the buoyancy of the hydrogel blanket (2.25 ± 0.23 g) was greater than the density of the cell culture medium (1.02 g/ml) [31], and the hydrogel blanket floated on the media (Fig. 1B^i^, C^i^). When the reservoir was filled with 4 g of cell culture media (total weight: 6.22 ± 0.23 g, total weight excluding buoyancy: 3.97 ± 0.23 g), the hydrogel blanket sank and applied pressure directly to the cells (when using 2% hydrogel gel, the pressure was 0.70 ± 0.02 kPa) (Fig. 1B^ii^-B^iv^ and C^ii^-C^iv^). At the same time, the hydrogel covered the cells to inhibit oxygen permeability, thus allowing the formation of a natural, hypoxic environment (Fig. 1C). We confirmed that the hydrogel blanket exerted a pressure on the cells and subjected them to a hypoxic environment. First, to assess whether pressure could be applied to the cells, the hydrogel blanket was placed on the chondrocytes cultured on a cell culture plate, and 4 ml of cell culture media was added to the reservoir of the insert. The pressure applied by the hydrogel blanket tools to the cells was measured considering the total volume, buoyancy, and cell culture media density. The pressure was calculated in terms of the weight per unit area. Therefore, the total weight of the hydrogel blanket tools was divided by the area, and the final pressure was obtained by subtracting the buoyancy of the hydrogel blanket tool. As a result, unlike the cells on which the hydrogel blanket was not applied (Fig. 1D, E), the cells covered by the hydrogel blanket exhibited compressed morphologies owing to the external pressure (Fig. 1F, G). Even when comparing the cell spreading area, it was found that the hydrogel blanket exerted pressure directly on the cells as the cell area expanded to 547 ± 334 µm^2^ in the case wherein cells did not use a hydrogel blanket and 1644 ± 545 µm^2^ in the case wherein the cells were covered by the hydrogel blanket. We tested the effect of the hydrogel blanket on oxygen permeability and assessed whether it was possible to control oxygen permeability according to the alginate concentration (Fig. S2). To achieve this, the following were prepared and oxygen permeations were measured (%): a) without the use of a membrane (open), b) with the use of a membrane (membrane), and c) with the use of a hydrogel blanket with a membrane (1, 2, 4, 6, 8% alginate gel) (Fig. 1H). Oxygen permeabilities of 100 ± 1.8% in the open case and 98.3 ± 1.9% in the membrane case were measured, but there was no statistical difference (*p* = 0.986) regardless of the presence or absence of the membrane. This implies that the membrane rarely affects oxygen permeation. However, the hydrogel blankets exhibited different characteristics. The oxygen permeability of the hydrogel blanket with 1% gel was 84.9 ± 8.9%, indicating an oxygen permeation inhibition of approximately 15.1%. The 2, 4, 6, and 8% gels yielded 64.1 ± 3.8%, 60.6 ± 4.8%, 51.9 ± 2.7%, and 46.3 ± 4.0% of oxygen permeability, respectively (Fig. 1I).

Gene expression comparisons confirmed whether the inhibition of oxygen permeation by the hydrogel blanket could provide cells with a low-oxygen culture environment. The transforming growth factor (TGF-β) is known to promote expression when chondrocytes are subjected to physical pressure [32]. Hypoxia-inducing factor 1 (HIF-1) is expressed when cartilage cells are exposed to hypoxic environments [25]. We compared and analyzed the expression of TGF-β and HIF-1 in cultured chondrocyte in a hydrogel blanket environment with western blotting methods. Through this, it was confirmed whether the hydrogel blanket culture provides physical compressive pressure and a hypoxic environment for cartilage cells. Unlike the control group (C) in which the hydrogel blanket was applied (P), both TGF-β and HIF-1 were expressed, thus confirming that the DCP culture with the hydrogel blanket provides both pressure and hypoxic environment to cells (Fig. 1J). The pressure applied by the hydrogel blanket to the cells was measured based on consideration of the total volume, buoyancy, and cell culture media density. At 2, 4, 6, and 8% alginate concentrations, the pressures were calculated as 0.70 ± 0.02 kPa, 0.73 ± 0.01 kPa, 0.75 ± 0.03 kPa, and 0.90 ± 0.18 kPa, which were proportional to the alginate concentration (Fig. 1K). Optimization of the hydrogel blanket was performed by comparing cell morphology and proliferation according to alginate concentration and culture time. To identify the hydrogel blanket conditions optimized for cell culture, the survival of chondrocytes was compared when the concentration of alginate was 0 (control), 2, 4, 6, and 8%, and was cultured for a total of four days in each environment. As a result, chondrocytes did not survive after four days at 4% and after two days at 6% or 8% of alginate concentrations. However, the cells that survived for more than four days were covered by the hydrogel blanket with 2% alginate (Fig. S3).

### Effect of biomimetic hydrogel blanket on the conservation of the intrinsic cellular properties (morphological analysis)

To determine the conservation effect of DCP culture with the hydrogel blanket, we compared cell morphology, which is known to be affected by the intrinsic cell properties [33]. A hypoxic environment was also evaluated as it is known to help maintain the intrinsic properties of chondrocytes [34]. In addition, by adding a hydrogel blanket in an artificial hypoxic environment, we tried to confirm whether a synergistic effect occurred when artificial hypoxia and DCP were combined.

**Fig. 2:**
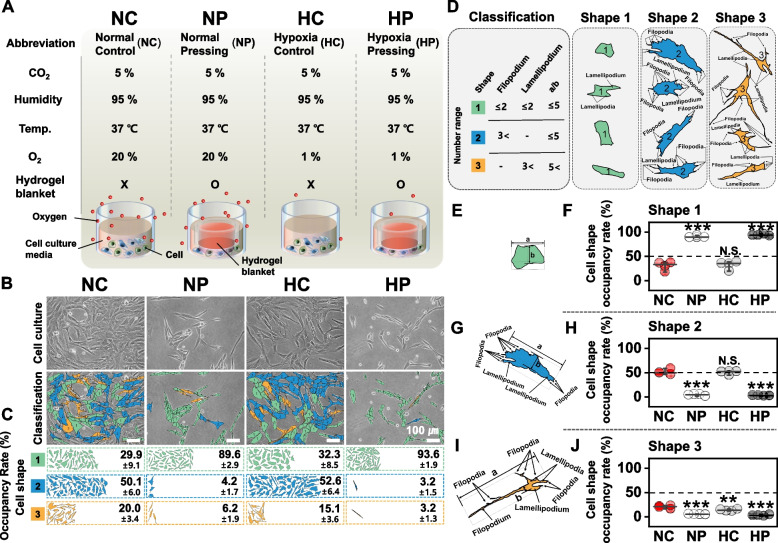
Changes in cell morphology according to the culture environment and numerical comparison of cell morphological changes using the classification criteria. (**A**) Cell culture conditions and schematic diagram: normal control (NC); a typical cell culture environment at 20% oxygen, 5% CO2, 95% or higher humidity, and 37℃. normal pressing (NP); hydrogel blanket applied in the same cultural environment as NC. hypoxia control (HC); hypoxic condition by artificially lowering the oxygen concentration to 1%. hypoxia pressing (HP); Artificially lowered oxygen concentration to 1% and additionally applied hydrogel blanket. (**B**) Phase-contrast microscopy images of chondrocytes cultured for 2 days in each environment of NC, NP, HC, and HP. Relative occupancy rate (**C**) according to the classification criteria of the cell shape (**D**). The representative morphology of shape 1 according to the cell shape classification criteria (**E**) and the proportion of shape 1 in each culture environment (**F**). The representative morphology of shape 2 according to the cell shape classification criteria (**G**) and the proportion of shape 2 in each culture environment (**H**). The representative morphology of shape 3 according to the cell shape classification criteria (**I**) and the proportion of shape 3 in each culture environment (**J**). In F-I, each graph represents the mean and standard deviation, and ANOVA analysis and Tukey’s post-hoc test were performed for statistical significance. N.S. (not significant), **P < 0.01, ***P < 0.001

A typical culture environment of CO_2_ (5%), humidity (95%), temperature (37℃), and O_2_ (20%) was defined as a normal control (NC) environment. By contrast, even if the CO_2_ (5%), humidity (95%), and temperature (37℃) conditions were the same, the culture environment in which the hydrogel blanket was applied in 20% O_2_ was defined as normal pressing (NP), the culture environment of 1% O_2_ was defined as hypoxia control (HC), and the culture environment in which the hydrogel blanket was applied to 1% O_2_ was defined as hypoxia pressing (HP) (Fig. 2A and Table S1).

The morphologies of chondrocytes cultured for two days in each environment (NC, NP, HC, and HP) were analyzed by classifying them according to the classification criteria (Fig. 2B,C, and Fig. S4, Table S2). “Shape 1” was defined as a cell with a relatively round shape (Fig. 2D, Shape 1 and Fig. 2E), similar to the morphology of normal chondrocytes with intrinsic properties maintained [35]. Similar to dedifferentiated chondrocytes that have lost their original properties [36], cells are classified as “shape 3” (Fig. 2D, shape 3, and Fig. 2I). A case in which similar to the fusion of the morphological features of shapes 1 and 3—was classified as “shape 2” (Fig. 2D, shape 2, and Fig. 2G). In NC and HC, shape 2 was the predominant cell type, with occupancy rates of 50.1 ± 6.0% and 52.6 ± 6.4%, respectively. Conversely, in NP and HP to which the hydrogel blanket was applied, shape 1 was the predominant cell type, and the occupancy rates were 89.6 ± 2.9% and 93.6 ± 1.6%, respectively (Fig. 2C). We compared the preservation effect of the hydrogel blanket on morphological chondrocyte changes according to the occupancies of shapes 1–3 and the environment (i.e., NC, NP, HC, or HP). Unlike in NC and HC, the proportion of shape 1, which is most similar to the original shape of chondrocytes, was significantly higher in NP and HP, to which the hydrogel blanket was applied (Fig. 2F). In addition, the occupancy of shape 3, which is similar to the dedifferentiated form, and that of shape 2, which is an intermediate form that changes from shape 1 to 3 owing to the loss of the original properties of chondrocytes, were also the lowest in NP and HP (Fig. 2H, J). These results show that the DCP culture method with the hydrogel blanket prevents morphological changes in chondrocytes.

### Effect of biomimetic hydrogel blanket on the conservation of intrinsic cellular properties (genetic analysis)

**Fig. 3:**
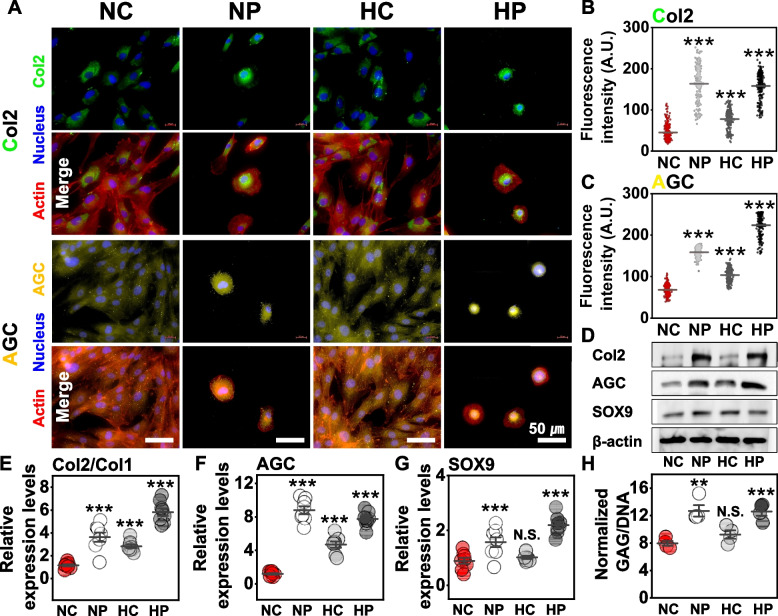
Effects of hydrogel blanket on the intrinsic properties of cells. (**A**) Immunofluorescence staining confocal microscopy results for Col2 and AGC expression of chondrocytes cultured for 2 days in each environment of NC, NP, HC, and HP. Results of analyzing statistical significance by quantifying the fluorescence intensities of Col2 (**B**) and AGC (**C**) in images obtained from a confocal microscope. (**D**) Western blot results of Col2, AGC, SOX9 genes and β-actin expressed in chondrocytes cultured in NC, NP, HC, and HP environments. Results of PCR analysis of Col2/Col1 (**E**), AGC (**F**), and SOX9 (**G**) expressed in chondrocytes cultured in each environment. (**H**) GAG/DNA amounts of chondrocytes cultured in each environment. In (B to C) and (E to F), each graph represents the mean and standard deviation, ANOVA analysis and Tukey’s post-hoc test were performed for statistical significance. N.S. (not significant), **P < 0.01, ***P < 0.001

The effect of the DCP culture environment in the case of the hydrogel blanket on maintaining the intrinsic properties of chondrocytes was confirmed at the genetic level. When chondrocytes lose their original properties, the expression of the type-II collagen (Col2) gene for the primary collagen type in articular cartilage [37] and aggrecan (AGC), a critical component of cartilage structure [38] are known to decrease [39]. We compared the expressions of Col2 and AGC using fluorescence staining and confocal microscopy (Fig. 3A). DCP cultured cells of NP and HP showed higher fluorescence intensity responses in the Col2 and AGC cases than those of NC and HC (Fig. 3B, C). When chondrocytes lose intrinsic properties, the expressions of Col2 and Col1 decrease and increase, respectively, thus resulting in a lower Col2/Col1 ratio [40]. In addition, transcription factor SOX-9 (SOX9) [41], which is essential for the differentiation of precursor cells into chondrocytes, GAG [42], the structural components of the extracellular matrix, and collagen fibers are known to decrease when chondrocytes lose their original properties. For this reason, the Col2/Col1 ratio, AGC, SOX9, and GAG were used for evaluating the intrinsic properties of chondrocytes [43]. As a result of Western blotting and polymerase chain reaction gene analysis, the relative expression levels of Col2/Co11, AGC, and SOX9 were higher in chondrocytes cultured in NP and HP environments than in cells cultured in NC and HC environments (Fig. 3E-G and Table S3). Interestingly, the expression levels of Col2/Col1 and AGC were higher in the artificial hypoxic culture environment than in the normal culture conditions, implying that the artificial hypoxic environment assists in the maintenance of the original properties of chondrocytes. Nevertheless, the DCP-cultured cells of NP and HP exhibited a more prominent conserving effect of intrinsic cellular properties than HC for chondrocytes (Fig. 3D-F). When NC and HC were compared to confirm the artificial hypoxic environment effect, HC cells yielded higher Col2/Col1 and AGC outcomes than NC cells, whereas SOX9 and GAG/DNA yielded no significant differences in HC and NC expression levels (Fig. 3G, H). This indicates that the hypoxic environment alone is not sufficient to maintain the original nature of the chondrocytes. Surprisingly, SOX9 and GAG/DNA as well as Col2/Col1 and AGC were promoted in NP and HP compared with NC and HC (Fig. 3G, H and Fig. S5, S6). From the above results, it was confirmed that the chondrocytes maintained their intrinsic properties in a superior way in the DCP culture environment to which the hydrogel blanket was applied compared with the artificial hypoxic condition.

### Recovery effect of biomimetic hydrogel blanket in cells which lost their intrinsic properties

Cells age and lose their original properties with the passage of time [44, 45]. When this happens, the restoration of their original properties is known as the “recovery effect” [46]. We investigated whether the use of the hydrogel blanket could affect the recovery of intrinsic properties, even in aged cells that lost their original properties. To this end, we compared the effects of DCP culture with the hydrogel blanket for each cell group at P2 (passage 2, in which cells were relatively young and retained their original properties), P4 (the intermediate stage of aging), and P9 (the older stage, in which cellular properties may be compromised), respectively, for two days.

**Fig. 4:**
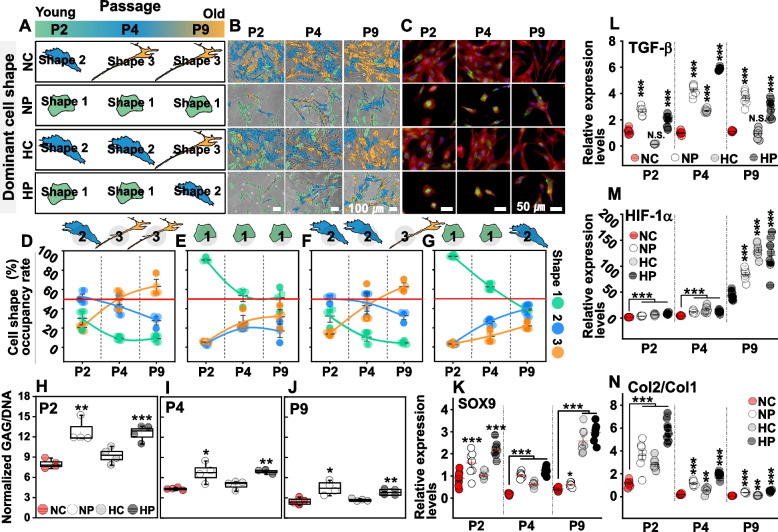
Conserving and recovery effects of intrinsic properties by DCP culture with the hydrogel blanket. Representative cell shape (**A**) and cell morphology (**B**) of relatively young to old chondrocytes in passage 2 (P2), passage 4 (P4), and passage 9 (P9) cultured in NC, NP, HC, and HP environments. (**C**) Col2 expression immunofluorescence staining confocal microscopy images of chondrocytes cultured in each environment. Cell shape occupancy rates according to passage and culture environment of NC (**D**), NP (**E**), HC (**F**), and HP (**G**). Results of comparative analysis of the amount of GAG/DNA after culturing cells of P2 (H), P4 (I), and P9 (J) in each environment of NC, NP, HC, and HP. PCR analysis results of SOX9 (K), TCF-β (L), HIF-1α (M), Col2/Col1 (N) expression of P2, P4, and P9 passage cells cultured in NC, NP, HC, and HP environments. Each graph of (H to N) represents the mean, standard deviation, and raw data. ANOVA analysis and Tukey’s post-hoc test were performed for statistical significance verification. N.S. (not significant), *P < 0.05, **P < 0.01, ***P < 0.001

As the passage of cells increased from P2 to P4 and P9, a distinct change in cell morphology was observed (Fig. 4A and Figs. S7, S8). In NC, shape 2 was the major cellular morphology in P2, but the occupancy rate of shape 2 decreased abruptly from P4, and that of shape 3 increased and became predominant (Fig. 4B, D and Fig. S9). Cells in HC also exhibited morphological changes similar to NC, but in NC, shape 3 was predominant in P4; by contrast, in HC, shape 3 became prevalent five generations later in P9 (Fig. 4B, F and Fig. S9). Cells in NP and HP to which the hydrogel blanket was applied exhibited markedly different morphological changes from NC and HC. In the NP condition, the occupancy rate of shapes 2 and 3 increased as the passage increased from P2 to P4 to P9, but shape 1 was the most prominent morphology (Fig. 4B, E and Fig. S9). HP showed similar characteristics to NP, but only in P2 and P4, and shape 1 was the main morphology, which changed to shape 2 in P9 (Fig. 4B, G and Fig. S9).

This result implies that in general cultures (such as NC), cells lose their original morphological characteristics after two generations (P2–P4); however, even those in the 9th generation can conserve their intrinsic morphology if the hydrogel blanket is applied. This further implies that applying the hydrogel blanket in a normal culture environment (NP) is more effective for the maintenance of the morphology than applying the hydrogel blanket in an artificial hypoxic environment (HP) (Fig. S9C).

We investigated (at the genetic level) whether the DCP culture with the hydrogel blanket affects the recovery of intrinsic properties even in cells which are already aging and losing their original properties. Fluorescence staining of Col2 fluorescence (green) was detected in P2 of NC, but fluorescence decreased as the generation increased to P4 and P9 (Fig. 4C). This trend was similar in the HC group (Fig. 4C and Fig. S10). In contrast, Col2 expression was observed in P2, P4, and P9 in NP and HP, and AGC exhibited a similar trend (Fig. 4C and Fig. S10). TGF-β, which is known to be expressed by physical pressure, was expressed at higher levels in NP and HP than in NC and HC, and was found to have little correlation with passage (Fig. 4I). HIF-1α was expressed in HC, NP, and HP in response to hypoxic environments and increased with increasing cell passage numbers (Fig. 4M). The expression of SOX9 was also promoted in cells cultured in the hydrogel blanket-applied environment and did not change significantly even with an increase in the number of generations (Fig. 4K). These results imply that the hydrogel blanket can not only maintain the original properties of primary chondrocytes but can also restore the original properties of cells which have already been lost owing to successive passages.

Interestingly, the chondrocytes of P9 in NP and HP yielded GAG/deoxyribonucleic acid (DNA) (Fig. 4H-J and Fig. S12, S13) and Col2/Col1 expression as high as those of P4 of NC (Fig. 4N and Fig. S11, S13). This suggests the possibility that the DCP culture method with the hydrogel blanket can recover properties (rejuvenation) for cells as young as approximately five generations, even in cells that have already lost their original properties.

### Conservation of intrinsic properties and recovery mechanisms of DCP culture

**Fig. 5:**
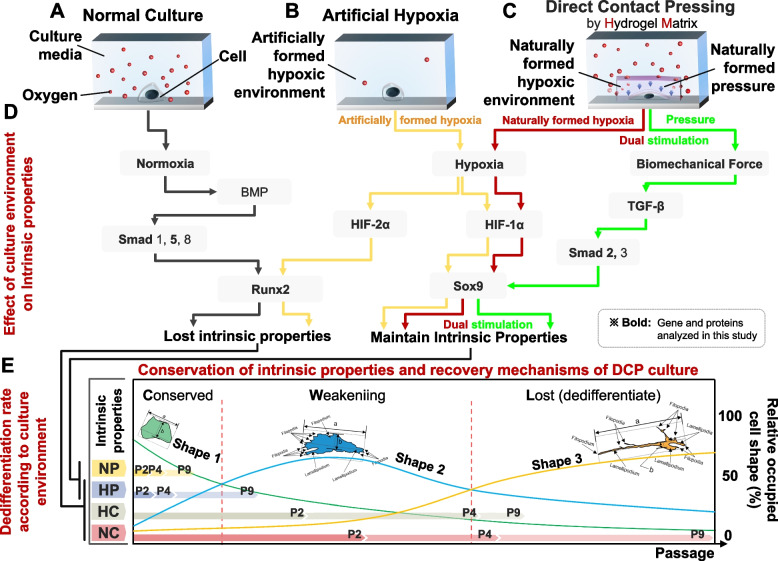
Mechanism of conserving and recovery of intrinsic properties of chondrocytes in DCP culture with the hydrogel blanket. Schematic diagram of the cell growth environment in each culture of normal culture (**A**), artificial hypoxia (**B**), and DCP culture with the hydrogel blanket (**C**). (**D**) Gene expression and signaling pathway steps of the cells cultured in each environment. Interrelationship diagram of culture environment, cell morphology, passage, and intrinsic properties. (**E**) Interrelationship diagram between culture environment, passage, cell type, and intrinsic properties

We aimed to investigate the mechanism associated with the maintenance and regeneration of the intrinsic properties of cells using a biomimetic hydrogel blanket. The stimuli received by cells in each culture environment, the various genes expressed by them (Table S3), and their signaling pathways (Table S4) are presented as a correlation diagram (Fig. 5). First, when cultured in a general environment such as NC (Fig. 5A), the bone morphogenetic protein (BMP), which constitutes a pivotal morphogenetic signal and orchestrates tissue architecture throughout the body [47, 48] is stimulated. In turn, it stimulates Smad 1, 5, and 8 (critically important for regulating cell development and growth) [49] associated with BMP. Smad promotes the expression of Runx2 (runt-related transcription factor 2, a key transcription factor associated with osteoblast differentiation) [50]. As a result, it is estimated that chondrocytes lose their original properties (Fig. 5D, E and Fig. S11, S13). In artificial hypoxic culture environments, such as HC (Fig. 5B), HIF-1α, a master transcriptional regulator of cellular and developmental responses to hypoxia [51], and HIF-2α, an essential mediator of the cellular oxygen-signaling pathway [52], are known to be expressed together. In this process, HIF-1α promotes the expression of SOX9 to assist the maintenance of the original properties of chondrocytes [53, 54], but the artificial hypoxic environment stimulates HIF-2α. HIF-2α promotes the expression of Runx2, leading to a decline in the intrinsic properties of chondrocytes [55, 56]. As a result, in the artificial hypoxic environment, SOX9, which is involved in maintaining the intrinsic properties of chondrocytes, and Runx2, which causes the loss of the original cellular properties, are expressed together, indicating that the effect of maintaining the original properties was insufficient (Fig. 5B,D,E and Fig. S11, S13).

Unlike general culture (NC) and artificial hypoxic environments (HC), in a DCP environment with the hydrogel blanket, cells receive direct pressure stimulation by the hydrogel (TGF-β), and also receive naturally formed hypoxic stimulation (HIF-1α) as the hydrogel surrounding the cell inhibits oxygen permeability (Fig. 5C). TGF-β expressed by pressure stimulation promotes the SOX9 (maintain the original properties of chondrocytes) [57, 58] through Smad 2, 3 (promote differentiation growth inhibition) [59, 60]. At the same time, HIF-1α expressed by hypoxic stimulation also promotes SOX9 [53, 61]. From the above results, the hydrogel blanket simultaneously applied direct pressure and hypoxic stimulation to cells, and these two stimuli promoted the expression of SOX9. It is presumed that the effect of maintenance of the intrinsic properties of cells was superior owing to this dual stimulation action (Fig. 5C-E and Fig. S11, S13). The reason why HP was unable to maintain the original cell properties compared with NP is attributed to the dual stimulation of SOX9 with HIF-1α and TGF-β by the hydrogel blanket (Fig. 5B-D). By combining the culture environment, cell morphology, and degree of dedifferentiation through genetic analysis of NC, NP, HC, and HP obtained heretofore, most NCs changed to shape 3 only in P4, and Col2/Col1, SOX9, and GAG were reduced. Thus, it should be noted that the rate of loss of the original shape and properties of chondrocytes was the fastest in NC. Conversely, in the NP environment wherein only the hydrogel blanket was applied, the effect of conserving the intrinsic properties of the cells was expected (Fig. 5E and Fig. S11-S13).

## Discussion

In this study, we developed a facile tool that creates a stamp-shaped hydrogel and implements a pressure and a low-oxygen environment without an external device. This biomimetic hydrogel blanket provides pressure and a hypoxic environment to cells using general-purpose cell culture equipment. The pressure and oxygen concentration can be adjusted through the hydrogel concentration to suit the characteristics of the cells and the purpose of the experiment. In addition, the hydrogel blanket can be used with commercially available tissue culture plates, such as 6-well plates. Therefore, it is possible to selectively apply and eliminate only the wells in which the cells are cultured in pressure and low-oxygen environments. To change the existing cell culture environment from normoxia (20% O_2_ concentration) [62] to hypoxia (1% O_2_ concentration) [63], a wait of more than 2 h is required to stabilize the gas in the cell incubator [64] or to prepare a separate hypoxic incubator for replacement [65, 66]. In addition, to apply pressure to the cells, financially burdensome hydrostatic pressure equipment [67, 68] or mechanical loading device [20, 69] are required.

In contrast, the hydrogel blanket creates a hypoxic and pressure environment within seconds at a low cost with the use of an existing incubator. In addition, the concentration of oxygen applied to the cells could be controlled by the hydrogel concentration, and the pressure applied to the cells could be easily regulated by the amount of the solution loaded into the insert’s reservoir. Because the hydrogel blanket uses a plastic insert as a mold, it could be easily handled with forceps and could be applied or removed at any time during the cell culture. Furthermore, the hydrogel blanket with surface topographical patterns could manipulate cell shape and alignment [70]. The hydrogel blanket effect associated with the maintenance of the intrinsic properties of cells was also superior to the use of an external device for the creation of an artificially low-oxygen environment. In an artificial hypoxic environment, genes that maintain the intrinsic properties and genes that dedifferentiate cartilage cells were expressed in sync. On the other hand, in DCP, both a hypoxic environment as well as physical pressure stimulation was provided to the cells. Consequently, the genes that maintain the inherent properties of cartilage cells are stimulated and doubly expressed in hypoxic and physical pressure environments. This double stimulation would have created a synergy effect in maintaining the intrinsic properties of the cartilage cells. Even when most of the original properties were lost owing to increased passage of the cells, the DCP culture could restore the original properties by five generations based on genes and by eight generations based on morphology. This demonstrates the possibility of applying the DCP culture technique to de-differentiate or re-differentiate cartilage cells that have lost their original properties.

## Conclusions

In this study, we developed a novel culture system that controls the pressure and oxygen concentration by covering the cells with only a simple blanket-type hydrogel. This technique does not require a special additional external machine and uses a conventional incubator to control the pressure and oxygen concentration applied to the cells. In fact, in the DCP culture system, the phenotype and genetic properties of chondrocyte are maintained. These results can be extensively applied to cell-based research, such as for the preservation and rebooting of intrinsic properties or response to oxygen and pressure environments. Therefore, the DCP culture with the hydrogel blanket is expected to be a novel, low-cost, easily manufacturable tool capable of and cell stimulation even when used in general-purpose incubators.

## Supplementary Information

**Additional file 1.**

## Data Availability

All data associated with this study are present in the paper and/or the Supplementary Materials.
