# Supplementary material for: Biomimetic hydrogel blanket for conserving and recovering intrinsic cell properties
Source: Biomater Res. 2022 Dec 13;26:78. doi: 10.1186/s40824-022-00327-w (PMC9746181; doi:10.1186/s40824-022-00327-w)

***Supplementary Material***

**Biomimetic hydrogel blanket for conserving and recovering intrinsic cell properties**

*Seung-Hoon Um^1,2,^* *^†^, Youngmin Seo^1,3, †^, Hyunseon Seo^1,4^, Kyungwoo Lee^1^, Sun Hwa Park^2^, Jung Ho Jeon^5,6^, Jung Yeon Lim^5^, Myoung-Ryul Ok^1^, Yu-Chan Kim^1,7^, Hyunjung Kim^8^, Cheol-Hong Cheon^9^, Hyung-Seop Han^1^, James R. Edwards^10^, Sung Won Kim^5,6*^, Hojeong Jeon^1,7,11*^*

^1^ Biomaterials Research Center, Biomedical Research Division, Korea Institute of Science and Technology (KIST), Seoul, 02792, Republic of Korea.

^2^ Laboratory for Biomaterials and Bioengineering, Canada Research Chair I in Biomaterials and Bioengineering for the Innovation in Surgery, Department of Min-Met-Materials Engineering, Research Center of CHU de Quebec, Division of Regenerative Medicine, Laval University, Quebec City, Quebec G1V 0A6, Canada.

^3^ R&D Institute, OID Ltd, Seoul 06286, Republic of Korea.

^4^ School of Medicine, Sungkyunkwan University, Suwon 16419, Republic of Korea.

^5^ Department of Otolaryngology-Head and Neck Surgery, The Catholic University of Korea, College of Medicine, Seoul, Korea.

^6^ Department of Biomedicine & Health Sciences, Department of Otolaryngology-Head and Neck Surgery, College of Medicine, The Catholic University of Korea, Seoul, Korea.

^7^ Division of Bio-Medical Science and Technology, KIST School, Korea University of Science and Technology, Seoul 02792, Republic of Korea

^8^ Division of Nursing, Research Institute of Nursing Science, Hallym University, Chuncheon, 24252, Republic of Korea

^9^ Department of Chemistry, Korea University, Seoul, 02841, Republic of Korea

^10^ Botnar Research Centre, Nuffield Department of Orthopaedics, Rheumatology and Musculoskeletal Sciences (NDORMS), University of Oxford, Oxford (OX3 7LD), United Kingdom

^11^ KU-KIST Graduate School of Converging Science and Technology, Korea University, Seoul 02841, Republic of Korea

* Corresponding author. Email: jeonhj@kist.re.kr, kswent@catholic.ac.kr

*^†^* These authors contributed equally: Seung-Hoon Um, Youngmin Seo


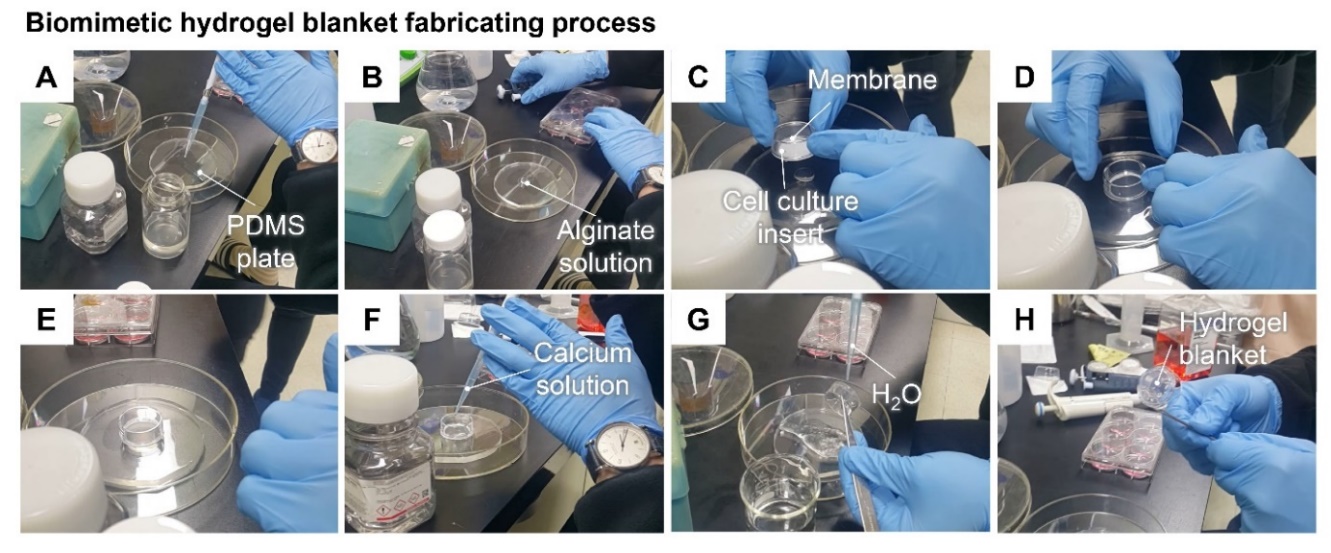


**Figure S1. Biomimetic hydrogel blanket assembling sequence and method.** (**A**) Prepare 2 wt % alginate solution, 1 M CaCl_2_ solution, and polydimethylsiloxane (PDMS) plates (10 cm diameter, 5 mm thickness). (**B**) 0.4 ml of alginate solution is dispensed on the PDMS plate. (**C**) Place the cell culture insert on the alginate solution. (**D**) Press gently by hand to spread the solution flat. (**E**) Check that no air bubbles have penetrated between the solution and the membrane (if bubbles are observed, repeat steps A to D). (**F**) Calcium solution was added to the alginate solution around the membrane using a pipette for gelation (1 min). (**G**) Distilled water is poured evenly over the gelled alginate for unreacted ions to be washed away. (**H**) After that, lift the cell culture insert using forceps and check whether the alginate gel sheet is formed flat under the membrane (If the gel surface is not formed flat, repeat steps A to F). The gelled alginate was immersed in phosphate buffered saline solution to remove excess calcium ions, and the solution was changed once a day for a total of 3 days and stored at 4℃ until the experiment. Blanket-shaped hydrogel was immersed in Dulbecco's Modified Eagle Medium (DMEM, Gibco) 3 hours before cell experiments and UV sterilized for 1 hour on a clean bench.


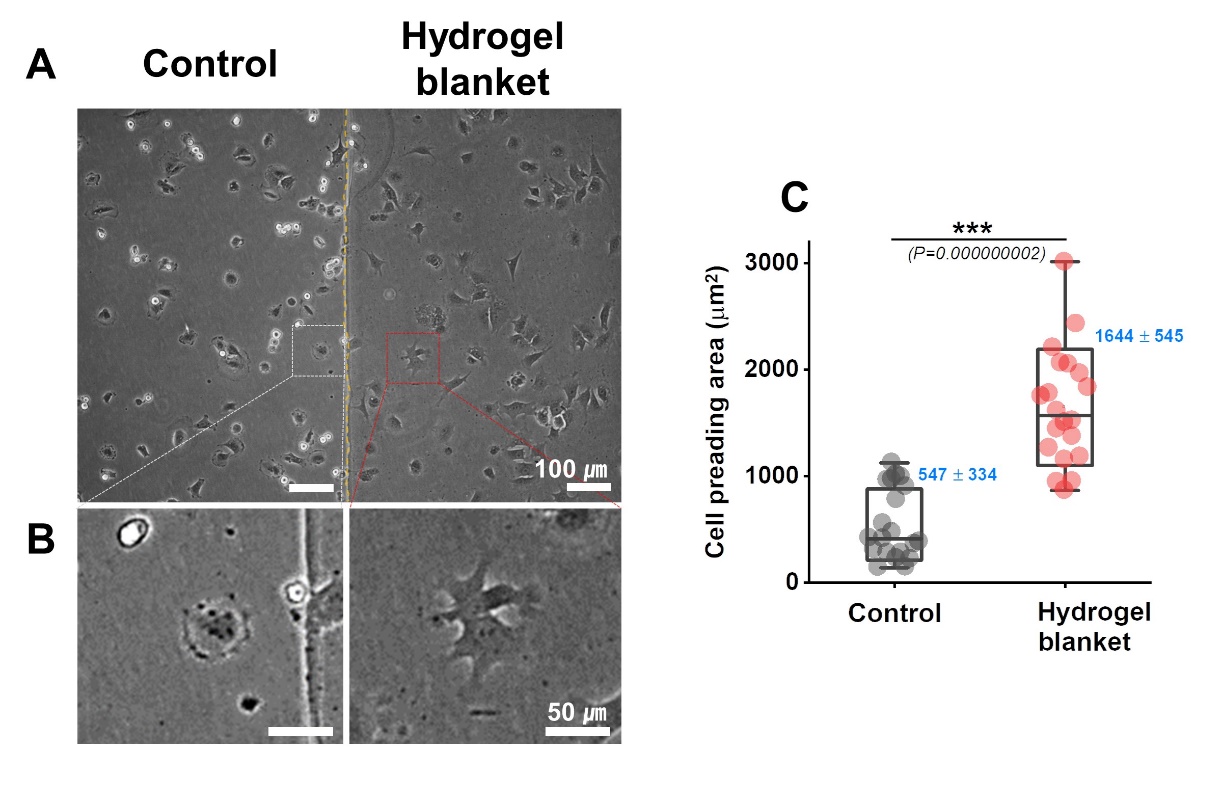


**Figure S2. Identification of chondrocyte stimulation effect of biomimetic hydrogel blanket.** (**A**) Changes in cell morphology according to presence (hydrogel blanket) or absence of hydrogel blanket (Control) in chondrocyte. After incubation for 1 hour on a cell culture dish, images were taken with a phase-contrast microscope immediately after hydrogel blanket application. (**B**) Phase-contrast microscopy high-magnification images of cells cultured in Control and hydrogel blanket environments. (**C**) Comparison of cell spreading area of chondrocyte with and without hydrogel blanket stimulation. The cell spreading area was measured and compared by using Image-J (image processing program, National Institutes of Health) of more than 20 cells obtained from a phase contrast microscope. Statistical significance was tested using a Student's t-test. *** P < 0.001.


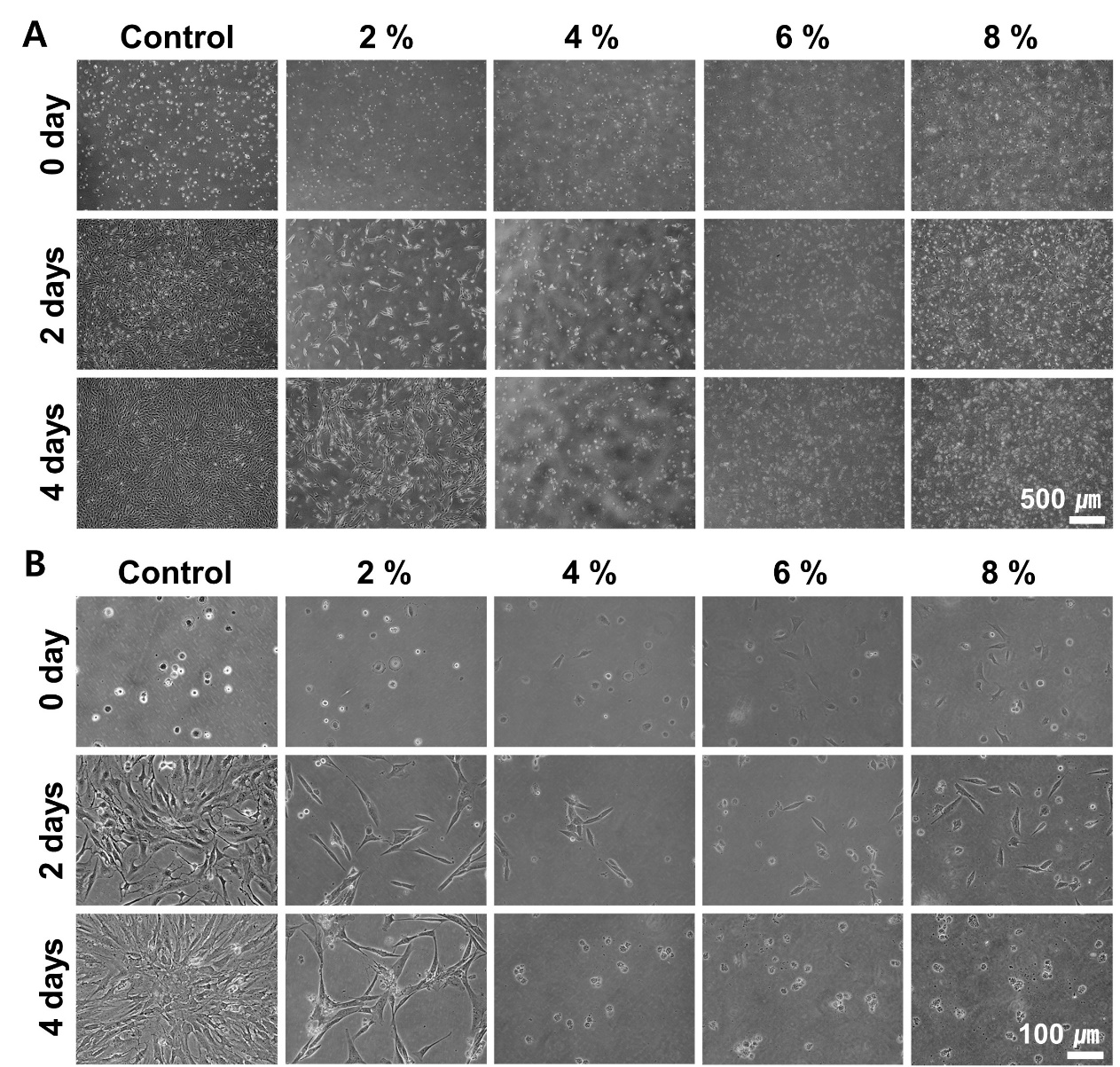


**Figure S3. Optimization of direct contact pressing (DCP) culture with a biomimetic hydrogel blanket using cell reaction according to alginate gel concentration.** Phase-contrast microscopy images of chondrocytes cultured for 2 days in a hydrogel blanket environment with 2, 4, 6, and 8% alginate gel concentrations. Culture without hydrogel blanket (Control), 10x magnification (**A**) and 20x magnification (**B**).

Table S1. Detailed culture environment table for NC, NP, HC, HP. CO_2_ concentration, humidity %, dissolved oxygen %, N_2_ concentration, temperature, presence or absence of hydrogel blanket - each culture environment table. ‘Hypoxia’ was defined as an intentional regulation of oxygen partial pressure in a cell culture system, and ‘Normoxia’ was defined as a state in which oxygen partial pressure was not artificially controlled. ‘Pressing’ was defined as a direct contact pressing culture with a biomimetic hydrogel blanket applied and ‘Control’ was defined as hydrogel blanket not applied in the culture environment.


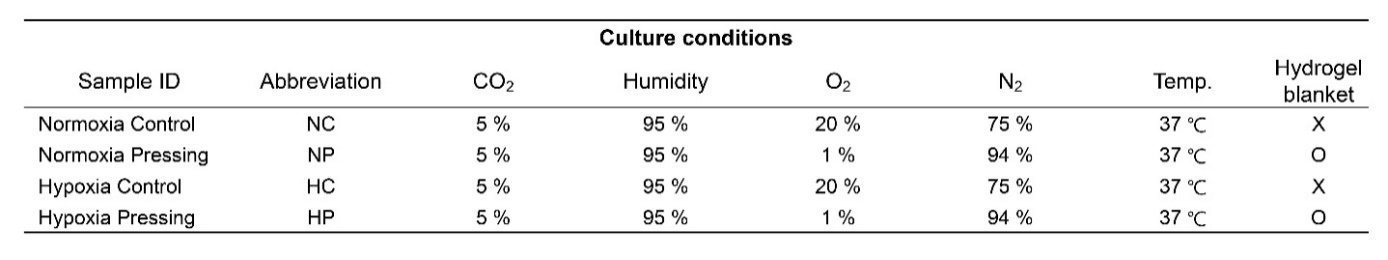


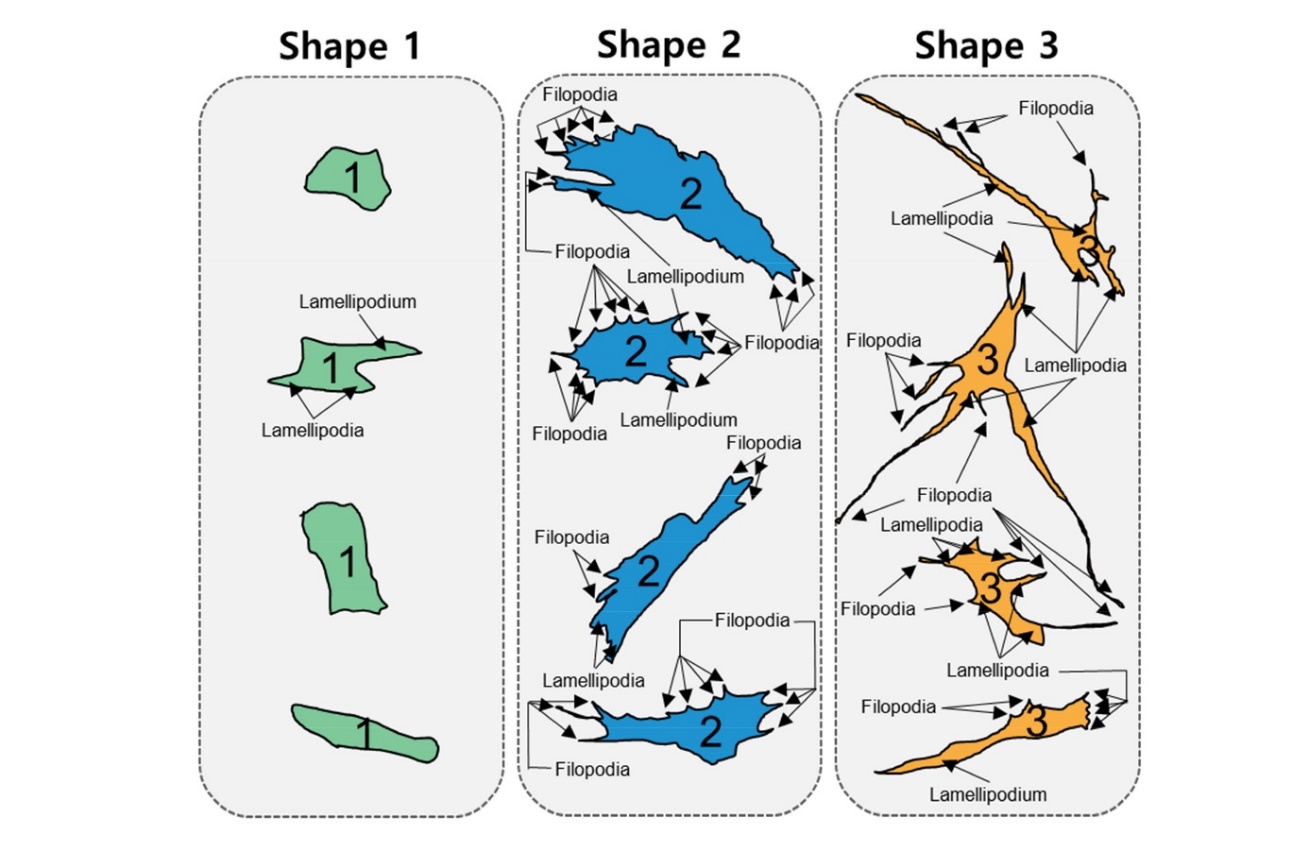


Figure S4. Criteria and examples of chondrocyte by morphology. The shape of chondrocytes is divided into three types according to the classification criteria: shape 1, shape 2, shape 3 and examples of each shape.

Table S2. Detailed criteria for classification of chondrocyte shape. The shape of chondrocytes is divided into three types according to the classification criteria: shape 1, shape 2, and shape 3. Definition and type classification criteria of 'length', 'width', lamellipodia, and filopodia required for chondrocyte type classification.


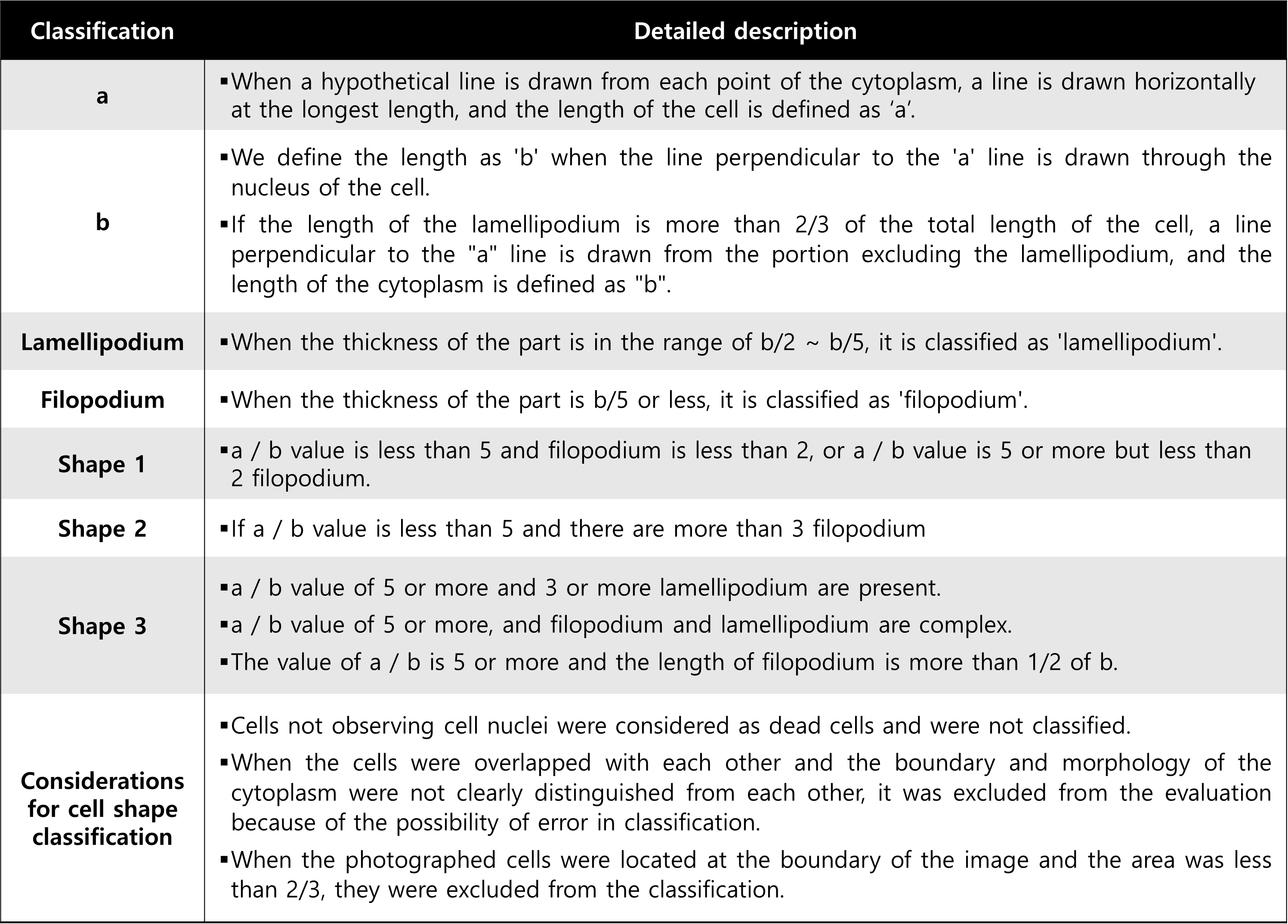


Table S3. Sequence list of primers used for gene analysis using real-time polymerase chain reaction (RT-PCR). Full names of Col1, Col2, AGC, SOX9, HIF-1α, TGF-β1, Smad2, Smad5, Runx2, and GAPDH. Reactive species, NCBI (National Center for Biotechnology Information) reference, sequence, and sequence length.


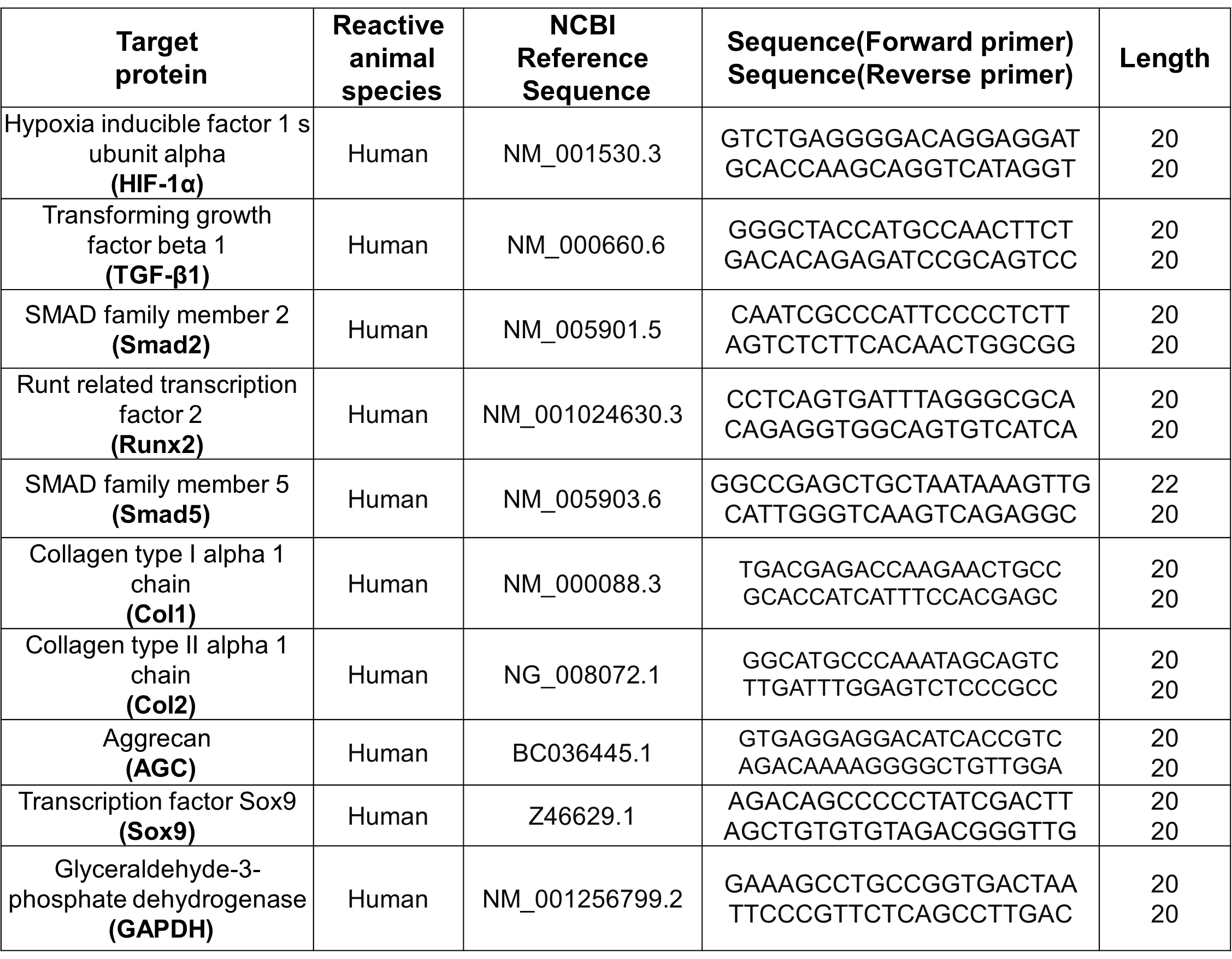


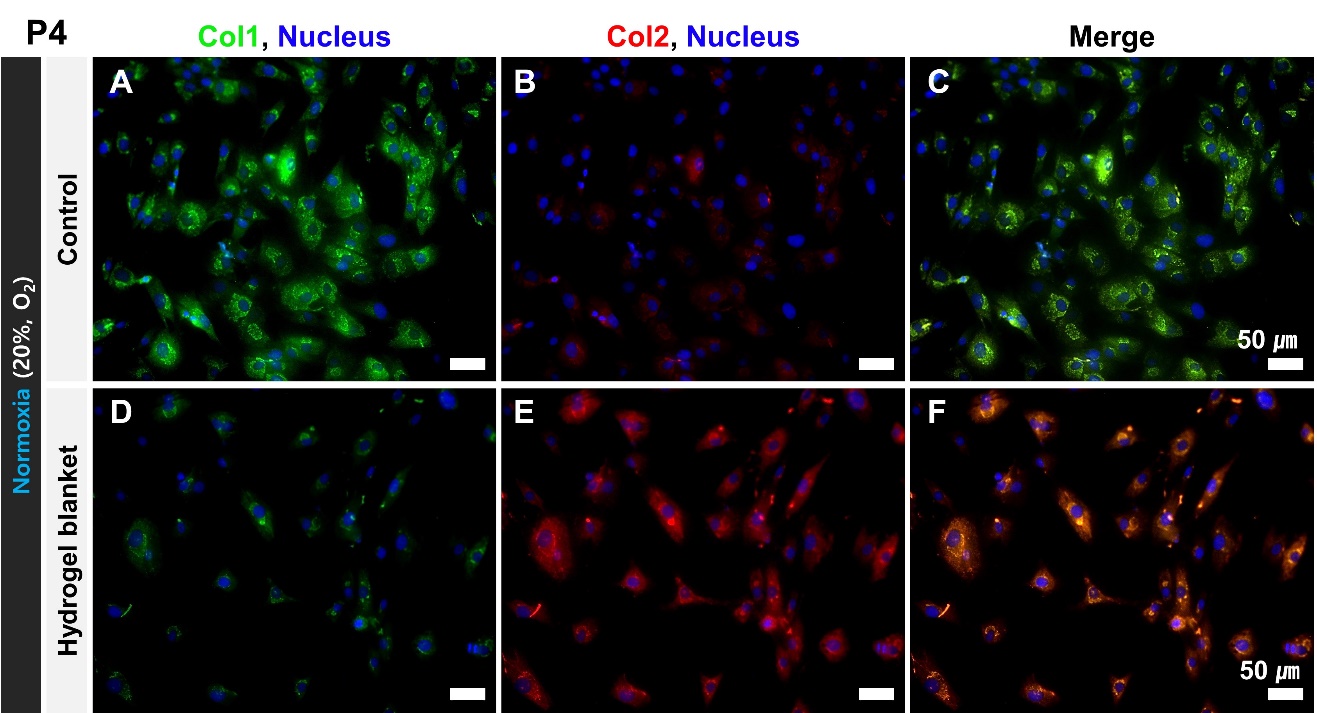


**Figure S5. Effects of Col1 and Col2 expression in chondrocytes according to direct contact pressing culture with a biomimetic hydrogel blanket application.** Col1 and Col2 expression images of chondrocytes cultured for 2 days in a culture environment without hydrogel blanket (Control) and a culture environment with hydrogel blanket (All images were taken using a confocal microscope after immunofluorescence staining). Col1 fluorescence staining images of chondrocytes cultured in Control (**A**) and hydrogel blanket (**D**) environments. Col2 fluorescence staining images of chondrocytes cultured in Control (**B**) and hydrogel blanket (**E**) environments. Col1 and Col2 fluorescence merged images of chondrocytes cultured in Control (**C**) and hydrogel blanket (**F**) environments. Chondrocytes from Passage 4, cultured in 20% normoxia environment, fluorescently stained; cell nucleus (blue), Col1 (green), Col2 (red).


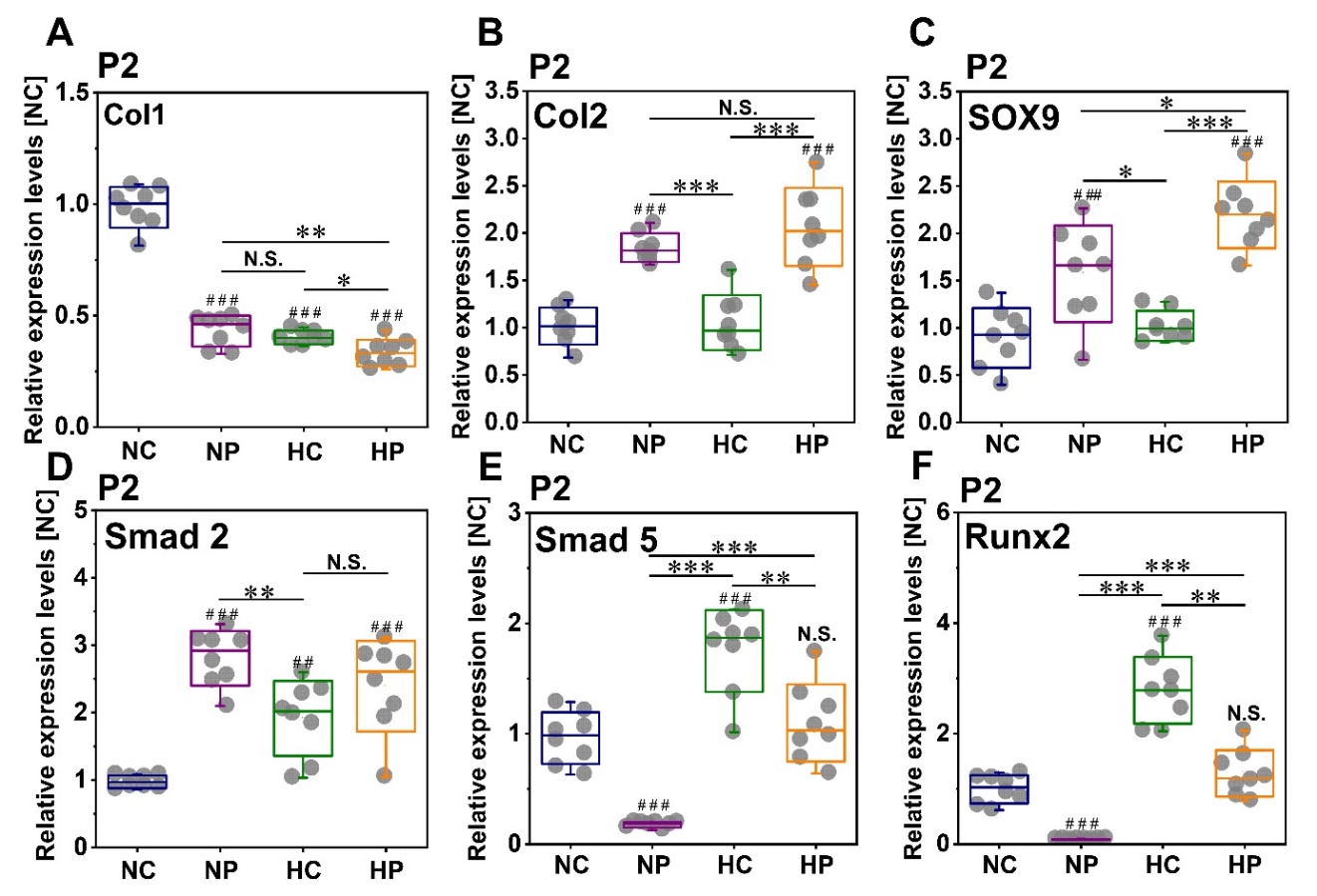


**Figure S6. Comparison of expression of genes related to chondrocyte intrinsic properties by culture environment.** Relative gene expression level of chondrocyte culture for 2 days in each external stimulus environment. Col1 (**A**), Col2 (**B**), SOX9 (**C**), Smad2 (**D**), Smad5 (**E**), Runx2 (**F**). All results were obtained from passage 2 chondrocyte. Statistical significance analysis was performed using one-way ANOVA test. Data represent mean ± s.d., *, **, *** indicate statistical significant difference when value compared to the each comparison group with * *p*<0.05, ** *P*<0.01, *** *P*<0.001. ^#^, ^##^, ^###^ indicate statistical significant difference when value compared to the ‘Normoxia Control (NC)’ condition with ^#^ *p*<0.05, ^##^ *P*<0.01, ^###^ *P*<0.001, (n=8).


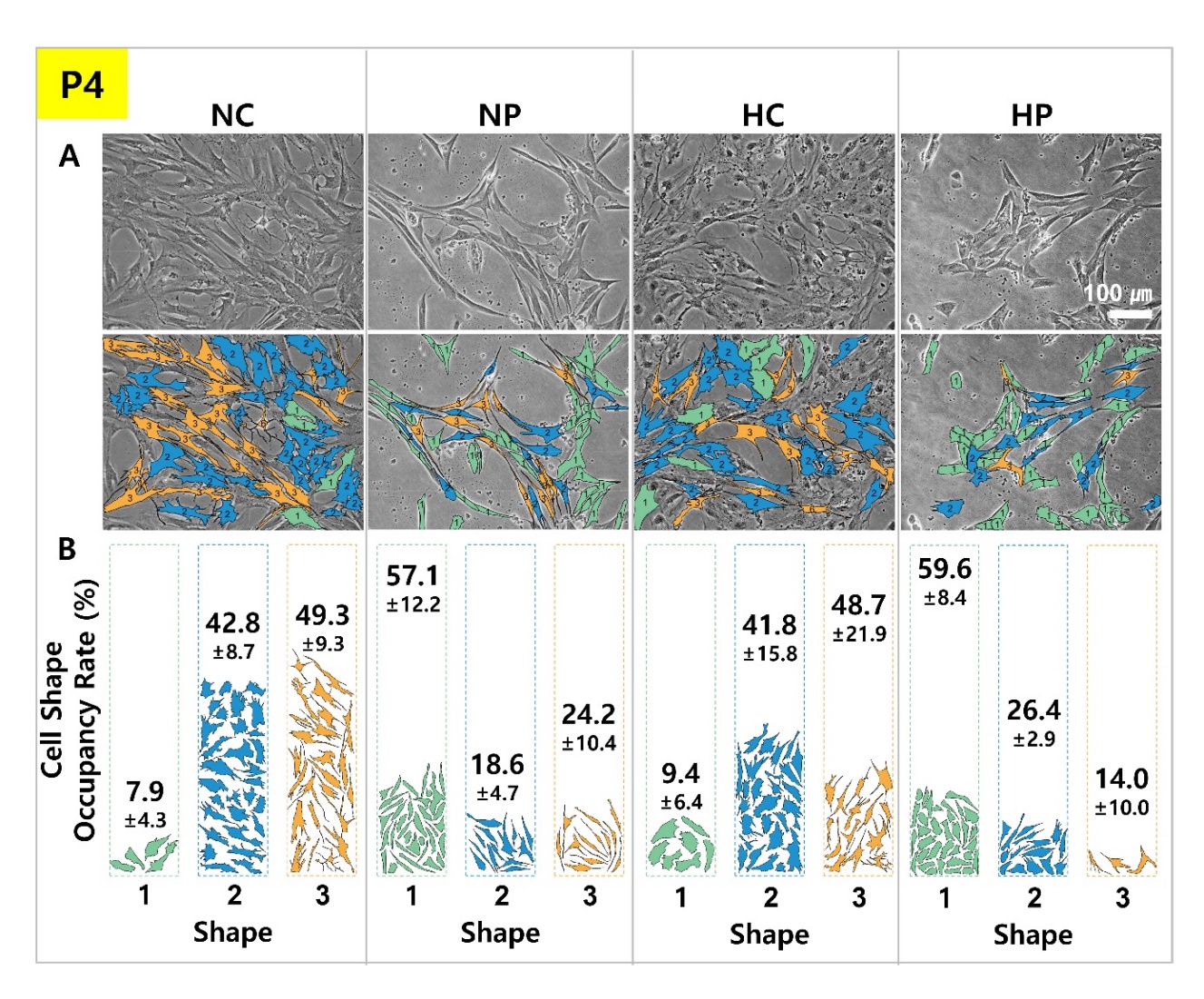


**Figure S7. Distribution of cell morphology in passage 4 by culture environment.** Cell morphology (**A**, upper row) and cell morphology classification results (**A**, lower row) by 'cell morphology classification criteria' after culturing chondrocytes for 2 days in each culture environment of NC, NP, HC, and HP. **B**, Cell shape occupancy rate analysis result by dividing the cell morphology observed in each environment into Shape 1, 2, and 3. The images of cells classified by shape are the size and shape of the actual cells obtained from a phase-contrast microscope, without modification. Numbers represent mean and standard deviation. The statistical significance test results are presented in ‘Figure S9’.


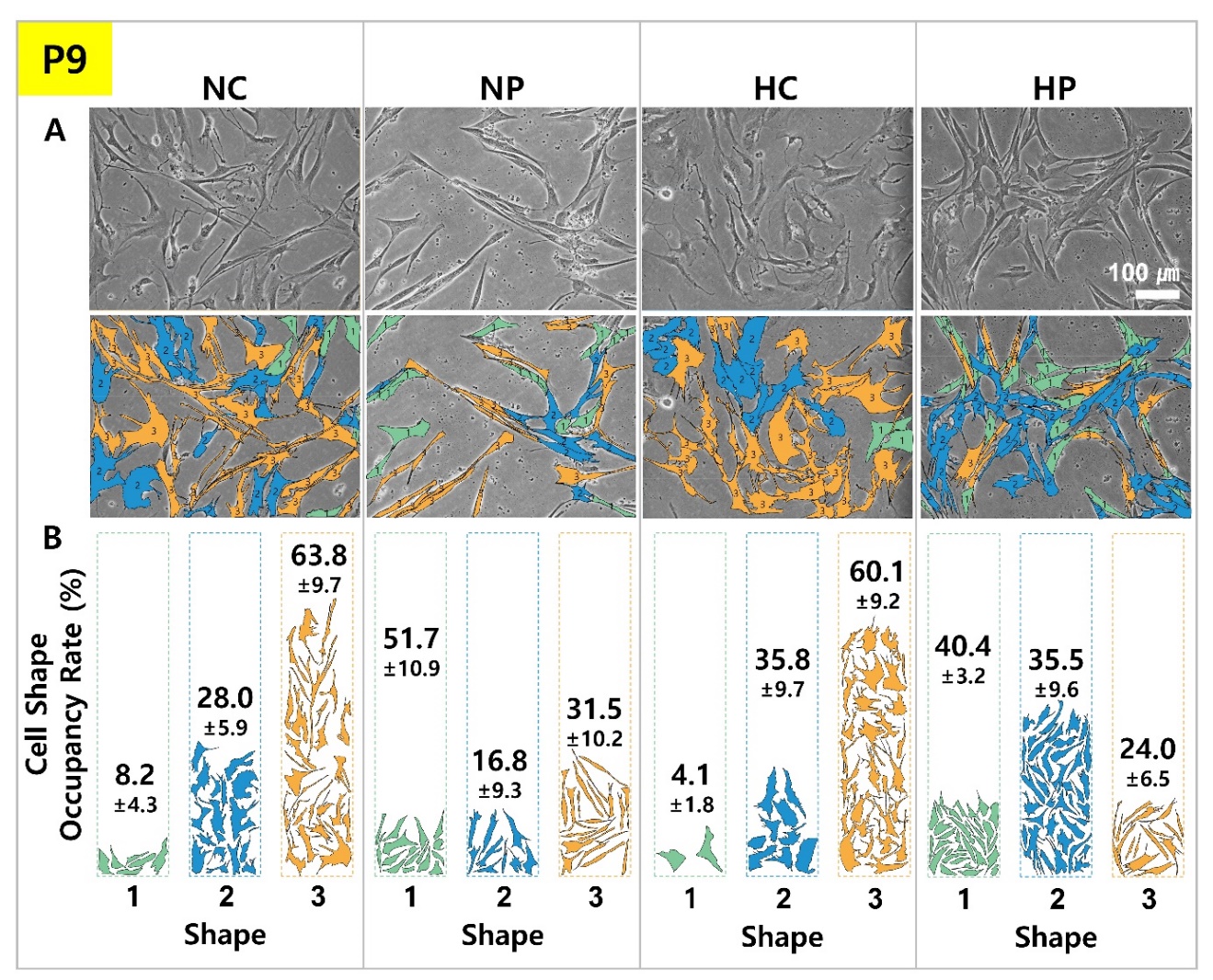


**Figure S8. Distribution of cell morphology in passage 9 by culture environment.** Cell morphology (**A**, upper row) and cell morphology classification results (**A**, lower row) by 'cell morphology classification criteria' after culturing chondrocytes for 2 days in each culture environment of NC, NP, HC, and HP. **B**, Cell shape occupancy rate analysis result by dividing the cell morphology observed in each environment into Shape 1, 2, and 3. The images of cells classified by shape are the size and shape of the actual cells obtained from a phase-contrast microscope, without modification. Numbers represent mean and standard deviation. The statistical significance test results are presented in ‘Figure S9’.


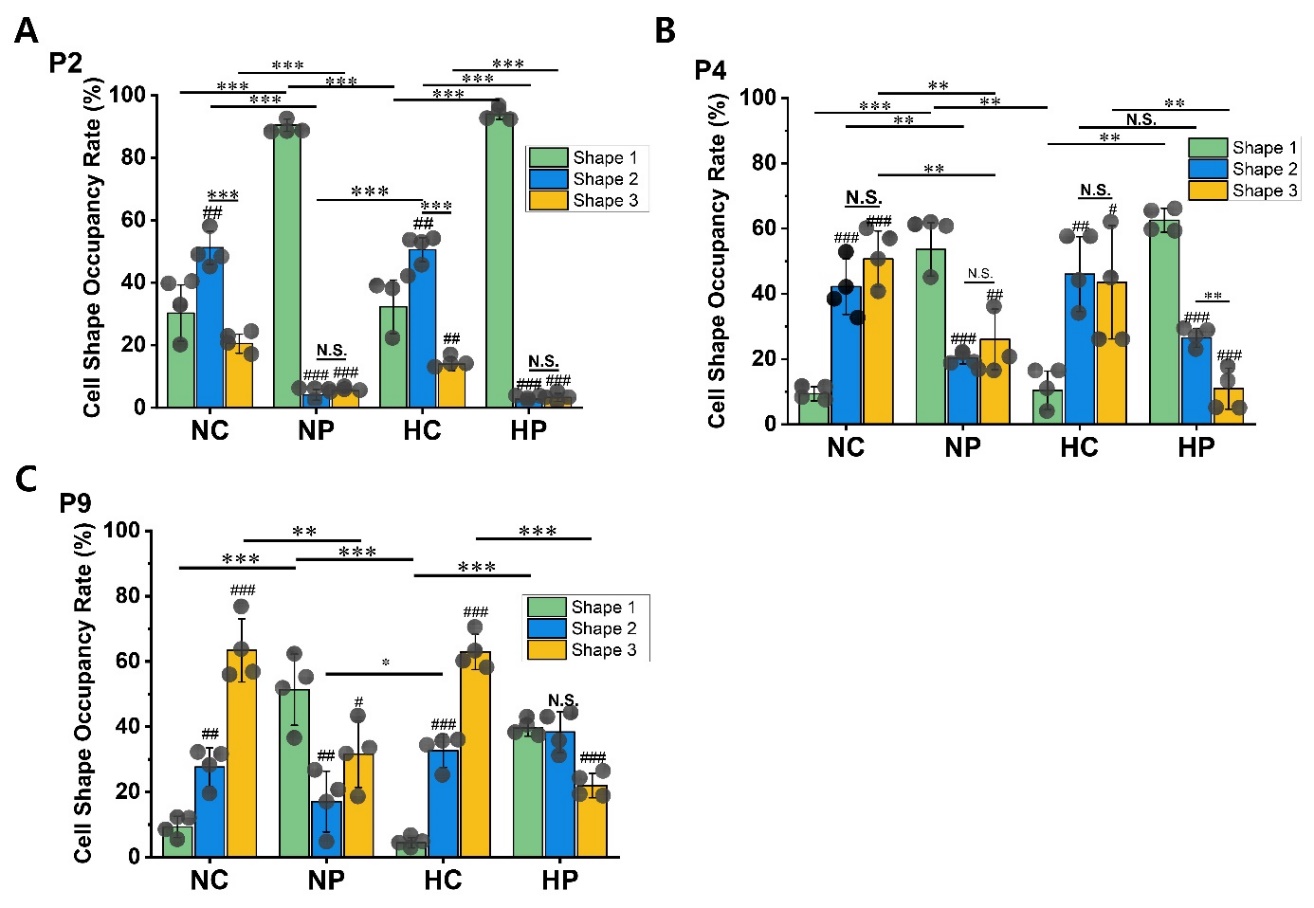


**Figure S9. Comparison of morphological changes according to passage and culture environment of chondrocytes.** The shape of chondrocytes is divided into three types according to the classification criteria: shape 1, shape 2, and shape 3. Morphological changes due to external environmental stimuli (NC, NP, HC, HP) and passage 2 (**A**), passage 4 (**B**), passage 9 (**C**). Each graph of a-c represents the mean, standard deviation, and raw data. ANOVA analysis and Tukey’s post-hoc test were performed for statistical significance verification. *, **, *** indicate statistical significant difference when value compared to the each comparison group with * *p*<0.05, ** *P*<0.01, *** *P*<0.001. ^#^, ^##^, ^###^ indicate statistical significant difference when value compared to the ‘Shape 1’ condition of each comparison group with ^#^ *p*<0.05, ^##^ *P*<0.01, ^###^ *P*<0.001.


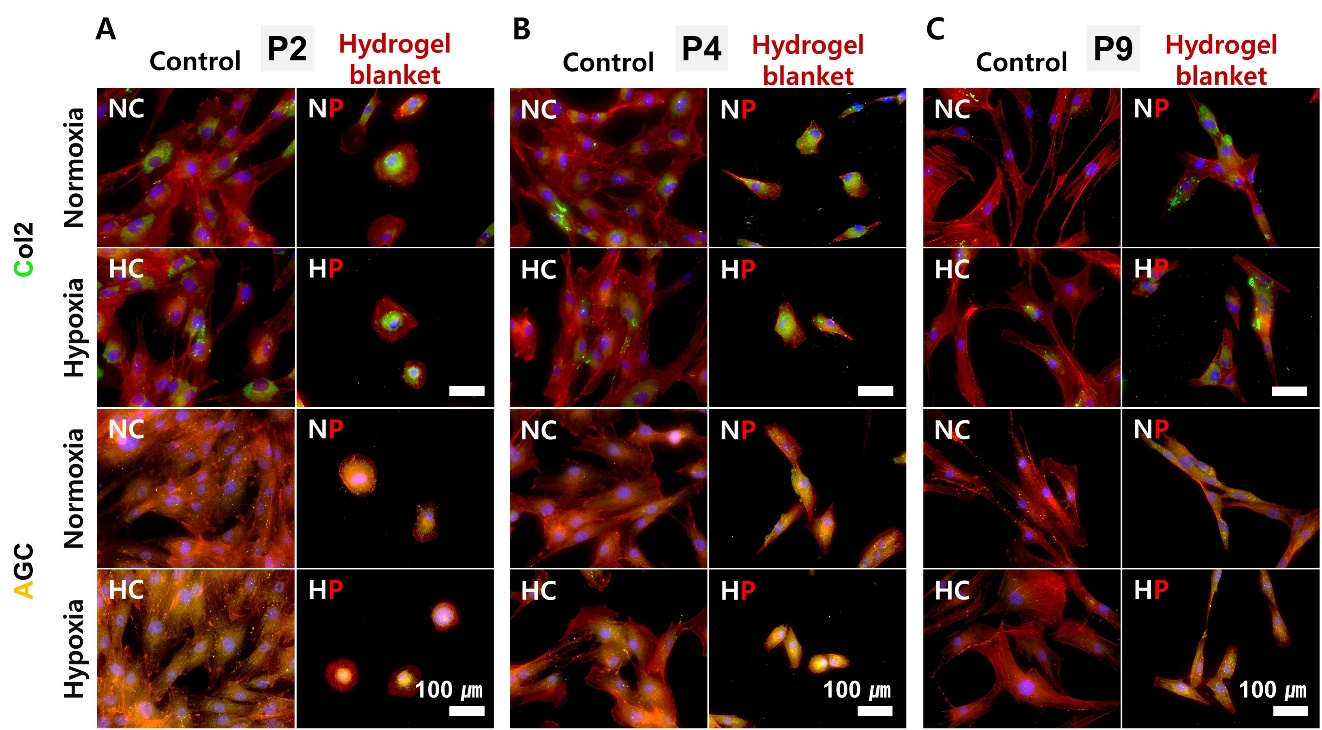


**Figure S10. Analysis of the effect of hydrogel blanket on Col2 and AGC recovering in relatively young and aged cells.** Col2 (green) and AGC (yellow) gene expression by hydrogel blank application from relatively young cells [passage 2 (**A**, P2), passage 4 (**B**, P4)] to relatively old cells [passage 9 (**C**, P9)]. Immunofluorescence staining was used and observed with a confocal microscope. Culture conditions and staining color: NC, NP, HC, HP cultured for 2 days in each environment, Normoxia; 20% O_2_, Hypoxia; 1% O_2_ nucleus (blue), cytoplasm (red).


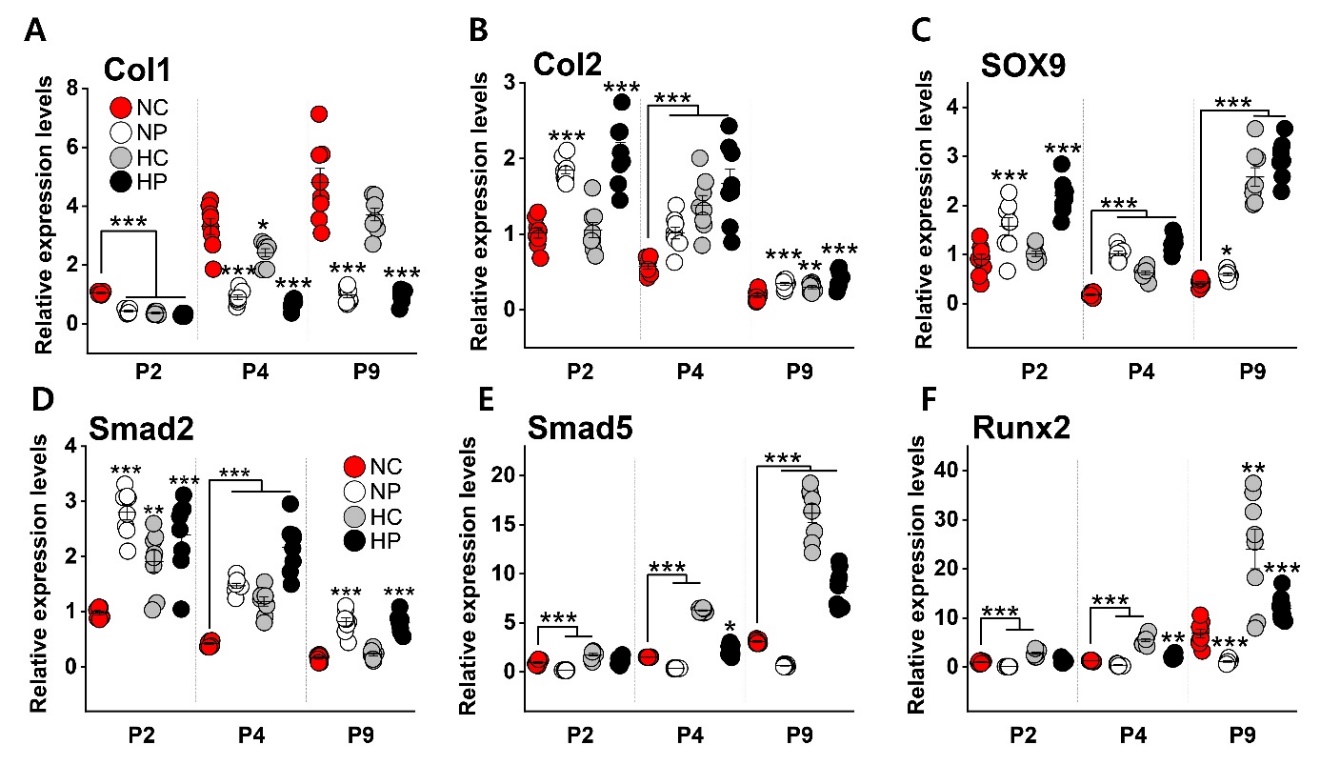


**Figure S11. Expression analysis of genes related to intrinsic properties of chondrocytes by passage and culture environment.** Relative expression levels of passage 2 (P2), 4 (P4), 9 (P9) chondrocyte cultures for 2 days in each external stimulus environment. Col1 (**A**), Col2 (**B**), SOX9 (**C**), Smad2 (**D**), Smad5 (**E**), Runx2 (**F**). Statistical significance analysis was performed using one-way ANOVA test. Data represent mean ± s.d. *, **, *** indicate statistical significant difference when value compared to the each ‘NC’ group with * *p*<0.05, ** *P*<0.01, *** *P*<0.001.


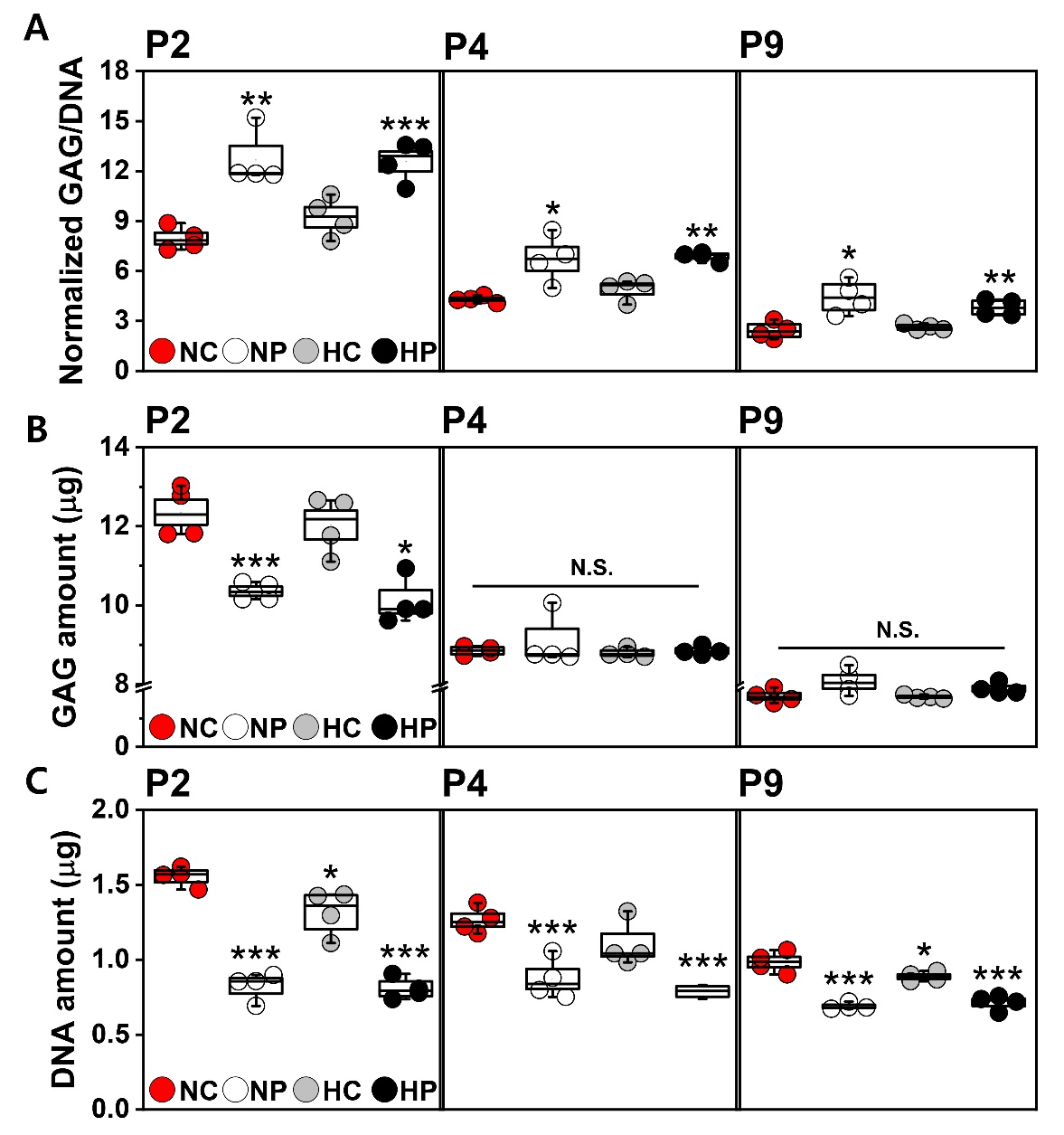


**Figure S12. Comparison of GAG amount according to culture environment and passage of chondrocytes.** (**A**) Comparison of normalized GAG/DNA amount according to environmental culture of NC, NP, HC, HP and passage 2, 4 and 9 chondrocytes. (**B**) Comparison of GAG amount (μg) of chondrocytes according to environmental culture of NC, NP, HC, HP and Passage 2, 4, 9. (**C**) Comparison of DNA amount (μg) according to environmental culture of NC, NP, HC, HP and Passage 2, 4, 9. Data represent mean ± s.d., one-way ANOVA was used for statistical significance analysis. *, **, *** indicate statistical significant difference when value compared to the ‘NC’ condition with * *p*<0.05, ** *P*<0.01, *** *P*<0.001.


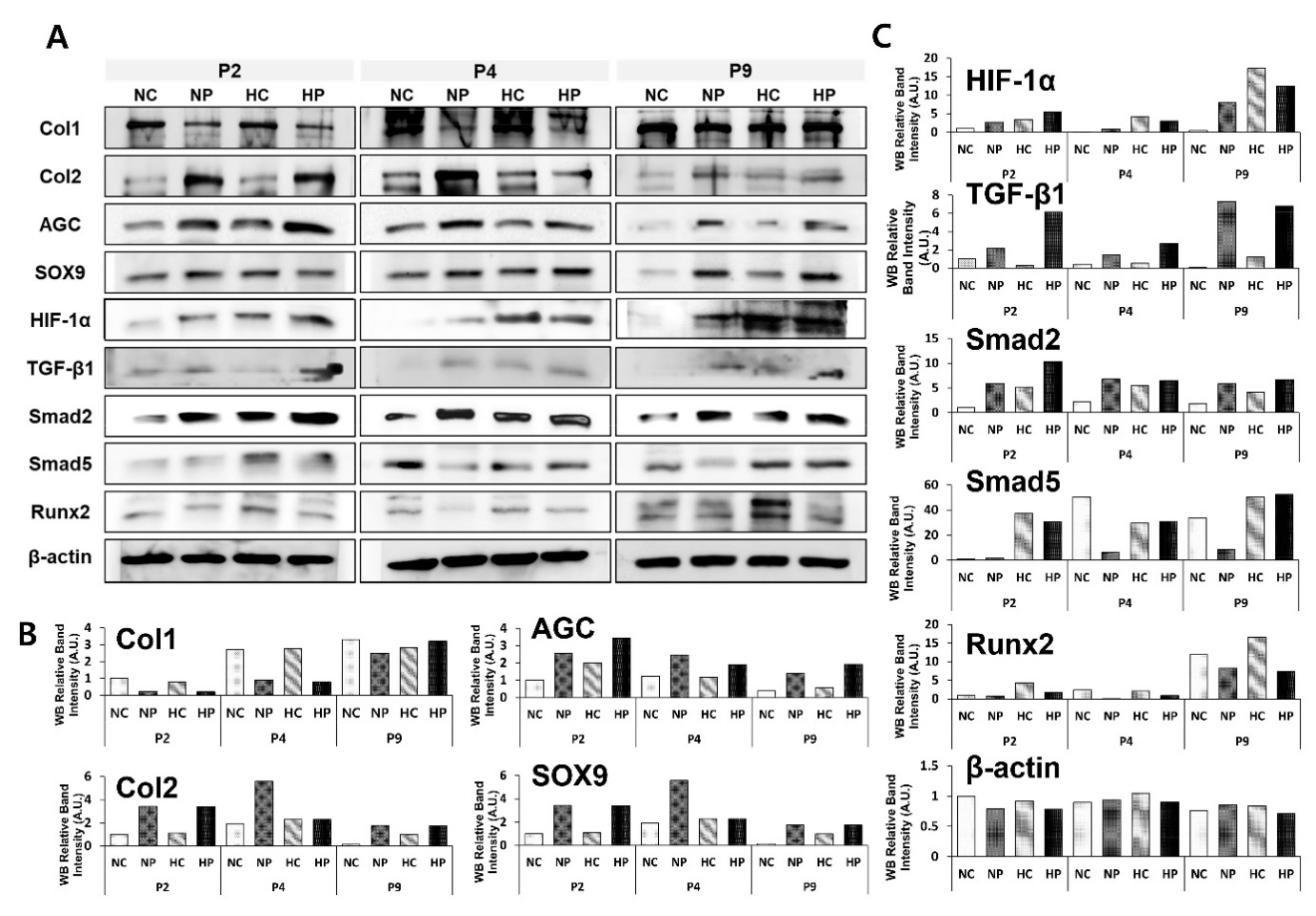


**Figure S13. The result of Western blot analysis of the effect of passage and culture environment on chondrocyte gene expression.** (**A**) Western blot results for the expression of Col1, Col2, AGC, SOX9, HIF-1α, TGF-β1, Smad2, Smad5, Runx2 and β-actin in Passage 2 (P2), Passage 4 (P4), Passage 9 (P9) chondrocytes cultured in NC, NP, HC, and HP environments. The result of quantifying the relative intensity of the protein band of Western blot: (**B**) Col1, Col2, AGC, and SOX9, (**C**) HIF-1α, TGF-β1, Smad2, Smad5, Runx2, and β-actin.

**Table S4. List of references, genes, and proteins for configuration signaling pathways (Figure 5) related to external environmental stimulation.**


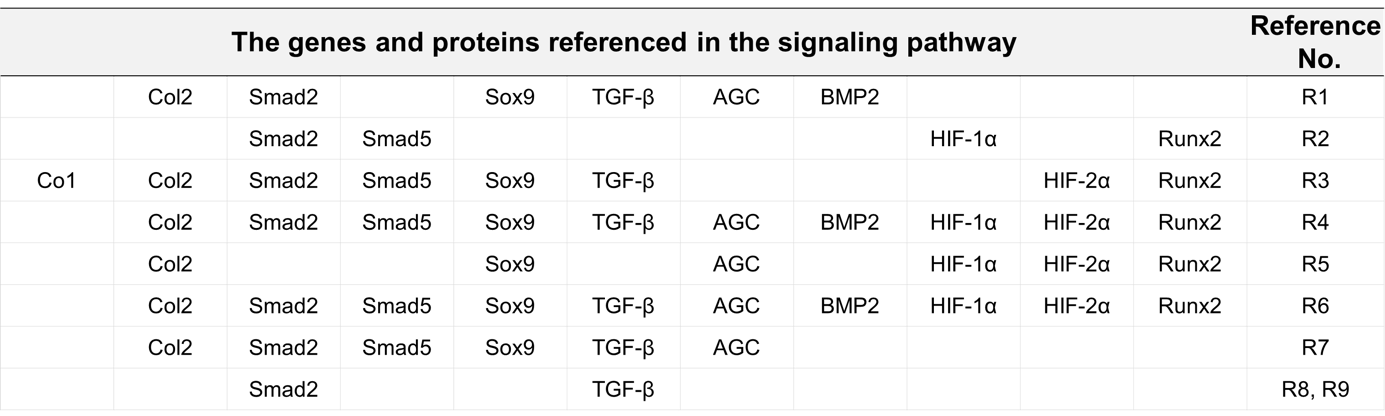


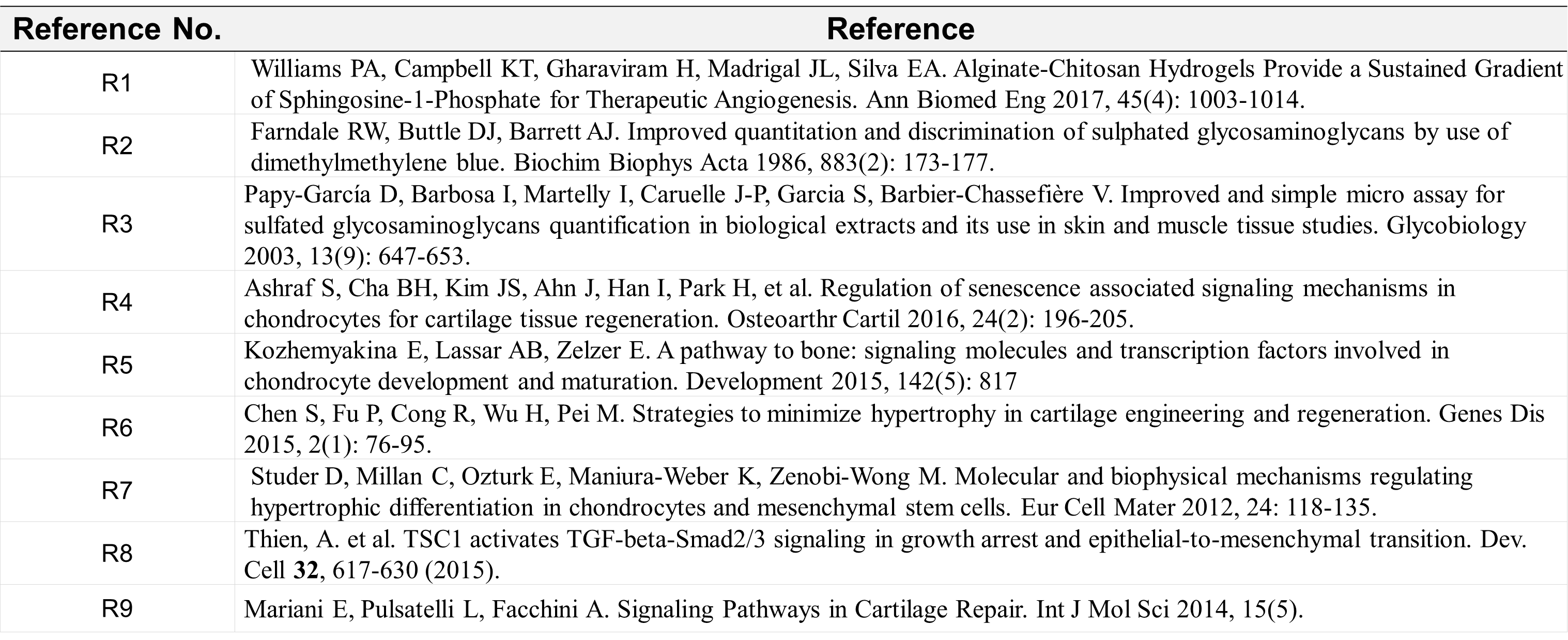

Supplement: Supplementary file 1 — Additional file 1. [file 40824_2022_327_MOESM1_ESM.docx]
